# Contaminant and Environmental Influences on Thyroid Hormone Action in Amphibian Metamorphosis

**DOI:** 10.3389/fendo.2019.00276

**Published:** 2019-05-14

**Authors:** Anita A. Thambirajah, Emily M. Koide, Jacob J. Imbery, Caren C. Helbing

**Affiliations:** Department of Biochemistry and Microbiology, University of Victoria, Victoria, BC, Canada

**Keywords:** thyroid hormone, environmental contaminant, endocrine disruptor, frog tadpole, metamorphosis, environmental factors, transcriptomics, genomics

## Abstract

Aquatic and terrestrial environments are increasingly contaminated by anthropogenic sources that include pharmaceuticals, personal care products, and industrial and agricultural chemicals (i. e., pesticides). Many of these substances have the potential to disrupt endocrine function, yet their effect on thyroid hormone (TH) action has garnered relatively little attention. Anuran postembryonic metamorphosis is strictly dependent on TH and perturbation of this process can serve as a sensitive barometer for the detection and mechanistic elucidation of TH disrupting activities of chemical contaminants and their complex mixtures. The ecological threats posed by these contaminants are further exacerbated by changing environmental conditions such as temperature, photoperiod, pond drying, food restriction, and ultraviolet radiation. We review the current knowledge of several chemical and environmental factors that disrupt TH-dependent metamorphosis in amphibian tadpoles as assessed by morphological, thyroid histology, behavioral, and molecular endpoints. Although the molecular mechanisms for TH disruption have yet to be determined for many chemical and environmental factors, several affect TH synthesis, transport or metabolism with subsequent downstream effects. As molecular dysfunction typically precedes phenotypic or histological pathologies, sensitive assays that detect changes in transcript, protein, or metabolite abundance are indispensable for the timely detection of TH disruption. The emergence and application of ‘omics techniques—genomics, transcriptomics, proteomics, metabolomics, and epigenomics—on metamorphosing tadpoles are powerful emerging assets for the rapid, proxy assessment of toxicant or environmental damage for all vertebrates including humans. Moreover, these highly informative ‘omics techniques will complement morphological, behavioral, and histological assessments, thereby providing a comprehensive understanding of how TH-dependent signal disruption is propagated by environmental contaminants and factors.

## Introduction

**Figure 1:**
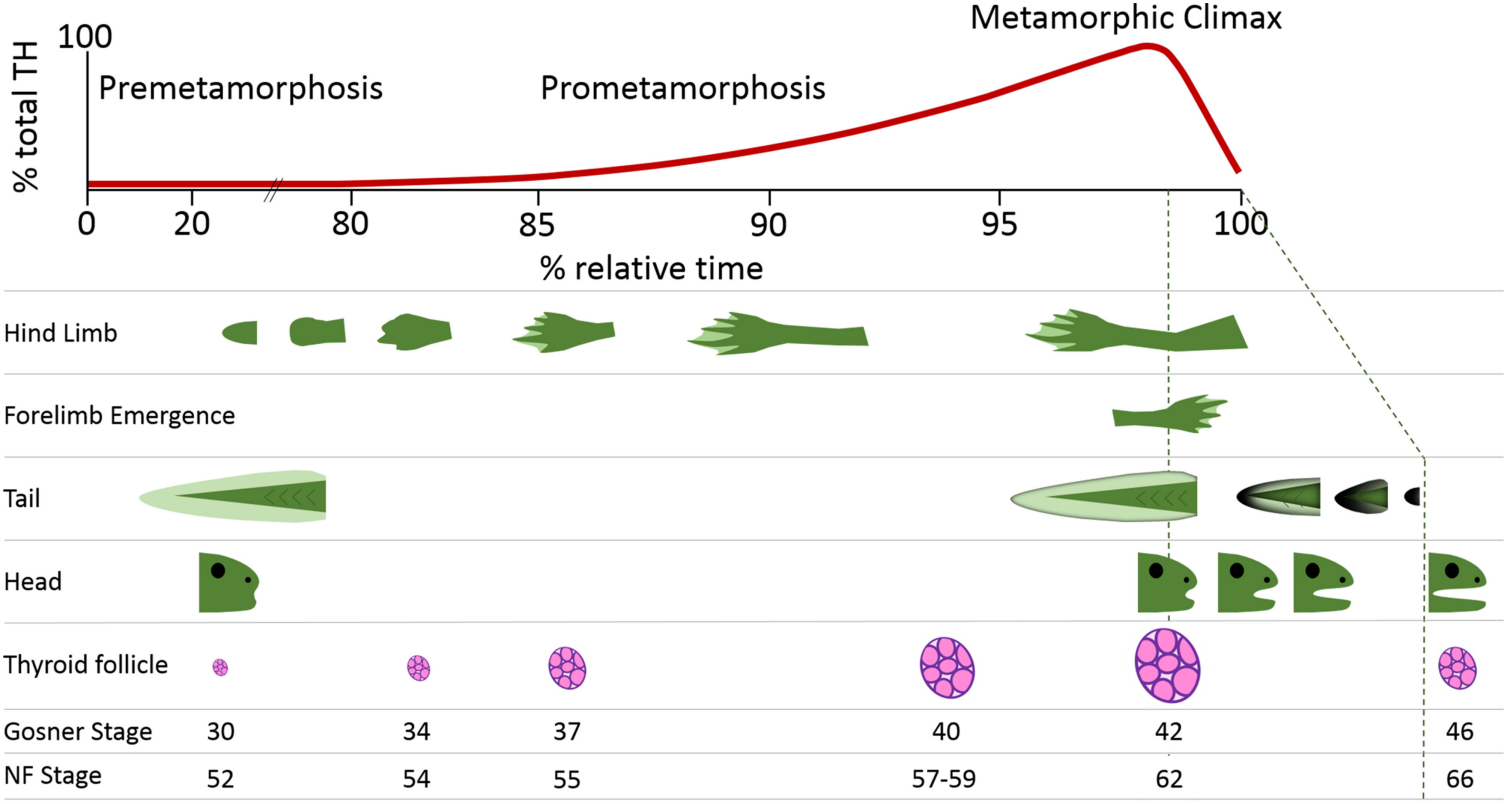
Thyroid hormone (TH) levels and key morphological hallmarks during frog postembryonic development. Amphibian metamorphosis is a postembryonic process driven by TH signaling. The free-swimming tadpole (0% relative time) has virtually undetectable levels of TH. The morphological changes that occur in the development of a tadpole to a juvenile frog (100% relative time) are inextricably aligned to internal rises in TH levels. These rising TH levels lead to progression through the stages of development, which can be seen through morphometric measurements including hindlimb development, forelimb emergence, tail regression, head shape changes, and thyroid follicle production. The Gosner and Nieuwkoop and Faber (NF) staging system comparisons are from Just (3).

Thyroid hormone (TH) signaling is a cornerstone of molecular events that mediate the profound morphological changes characteristic of early vertebrate development (1). The obligate requirement for TH is perhaps best exemplified by metamorphosing anuran amphibians for which the essential stimulation by TH initiates transitions from larval to juvenile stages under conducive environmental conditions (2). Amphibians undergo complex and comprehensive morphological changes as functionally athyroid premetamorphic tadpoles progress through prometamorphosis (with concurrent, increasing endogenous TH levels) and into juvenile frogs after metamorphic climax (Figure 1) (4). These changes encompass the coordinated maturation and remodeling of organs, *de novo* generation of limbs, regression of the tail, and the consequent alteration in behavior, diet, and niche as most aquatic tadpoles develop into more terrestrial-dwelling frogs (Figure 1) (5).

**Figure 2:**
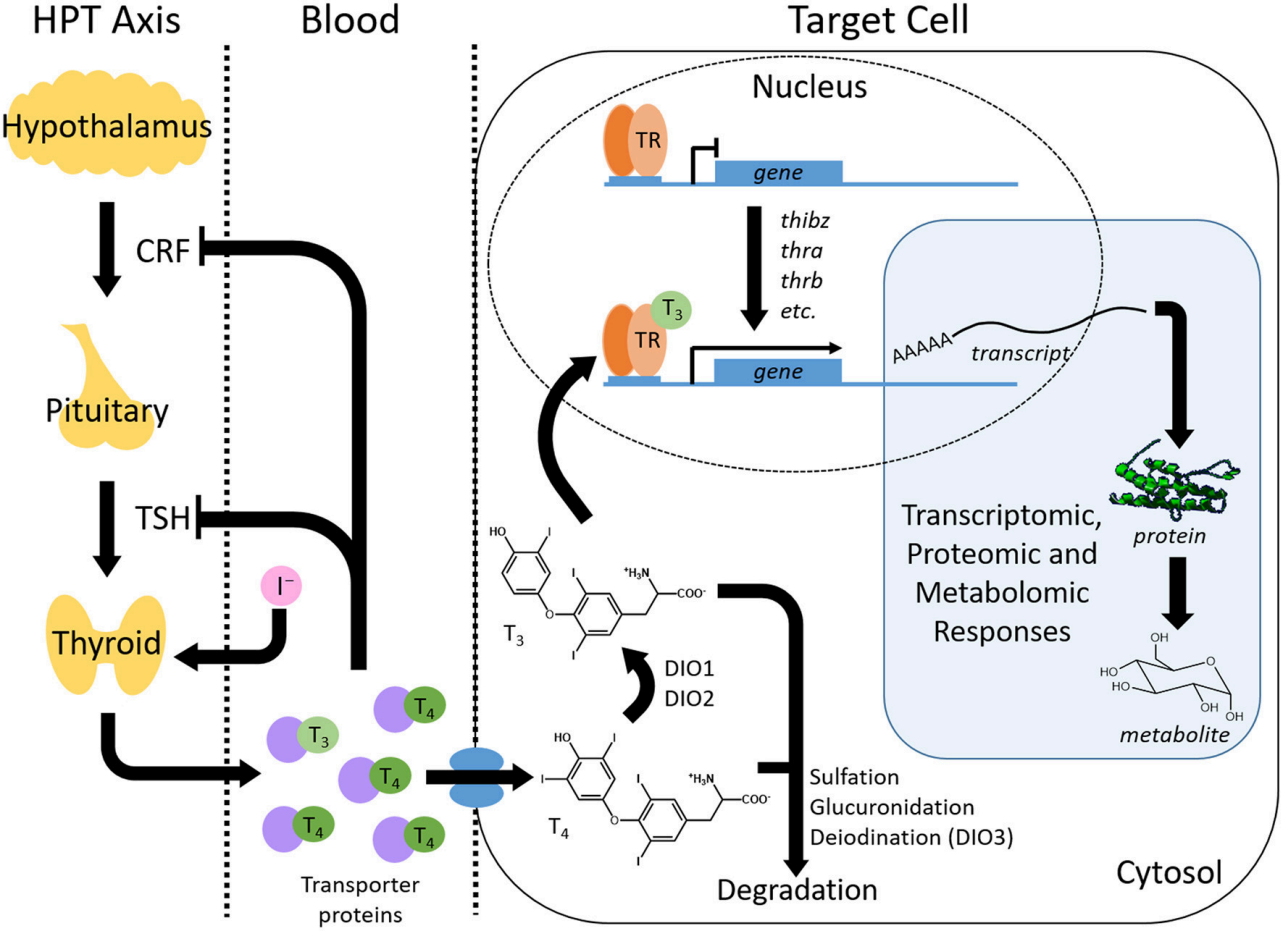
Overview of thyroid hormone (TH) production, transport, activity and regulation. The thyroid hormone signaling pathway involves a complex interplay between TH synthesis, transport, signal transduction, and catabolism. TH is synthesized within the hypothalamus-pituitary-thyroid (HPT) axis where the pituitary is stimulated to release thyroid stimulating hormone (TSH) by corticotropin releasing factor (CRF) from the hypothalamus. TSH induces the production of thyroxine (T_4_) and, in lesser amounts, triiodothyronine (T_3_) from the thyroid gland. The production of TH self-regulates through a negative feedback loop that inhibits further CRF and TSH production. TH travels through the blood via transporter proteins to peripheral tissues where it is imported into target cells. Here, T_4_ is converted to T_3_ through deiodinases (DIO), although T_4_ can bind to receptors as well. Binding of THs to TH nuclear receptors (TR) leads to the activation of TH response genes. This change in transcript abundance results in downstream proteomic and metabolomic responses that produce the phenotypic changes resulting from the TH signal. The TH signal is also regulated within the cell by catabolism that includes processes such as sulfation, glucuronidation and deiodination.

TH production is controlled by the hypothalamic-pituitary-thyroid (HPT) axis (Figure 2). The hypothalamus stimulates the pituitary with corticotropin releasing factor (CRF) to release thyroid stimulating hormone (TSH). TSH promotes the synthesis of TH in the follicular cells of the thyroid gland (2). The central dogma of TH signaling is that the newly synthesized prohormone thyroxine (T_4_) is transported from the thyroid gland by transporter proteins (e.g., transthyretin). Once at the destination peripheral tissue, T_4_ is converted into its more active form, 3,3′,5-triodothyronine (T_3_), by the enzymatic activity of deiodinases (Figure 2). Additionally, the bioactivity of T_4_, without conversion, has recently been demonstrated (6–9). TH binds its TH receptors (TRs), TRα, and TRβ, which are constitutively bound to cognate receptor elements that regulate genes sensitive to TH. Metamorphosis is initiated in anurans upon TH production, which stimulates gene expression cascades and subsequent proteomic and metabolomic alterations (Figure 2) (10, 11). TH metabolism is regulated through various enzymatic activities (glucuronidation, sulfation, and deiodination), which can target the hormone for degradation and thereby modulate TH activation of gene expression (Figure 2). For more detailed descriptions of thyroid hormone production, activity, and metabolism, the reader is encouraged to consult the following publications and the references therein (2, 12–15).

The spatiotemporal control of TH-dependent molecular and physiological activities during metamorphosis is particularly sensitive to abiotic and xenobiotic perturbations. Although the mechanism of molecular interference is not known for most adverse exposures, disruption can potentially target any aspect of TH synthesis, activity, and metabolism (Figure 2). Such disruptions include the exposure of premetamorphic tadpoles to exogenous TH, which results in a precocious induction of metamorphosis that can be exploited to experimentally assess toxicant perturbations during this developmental period (2).

**Figure 3:**
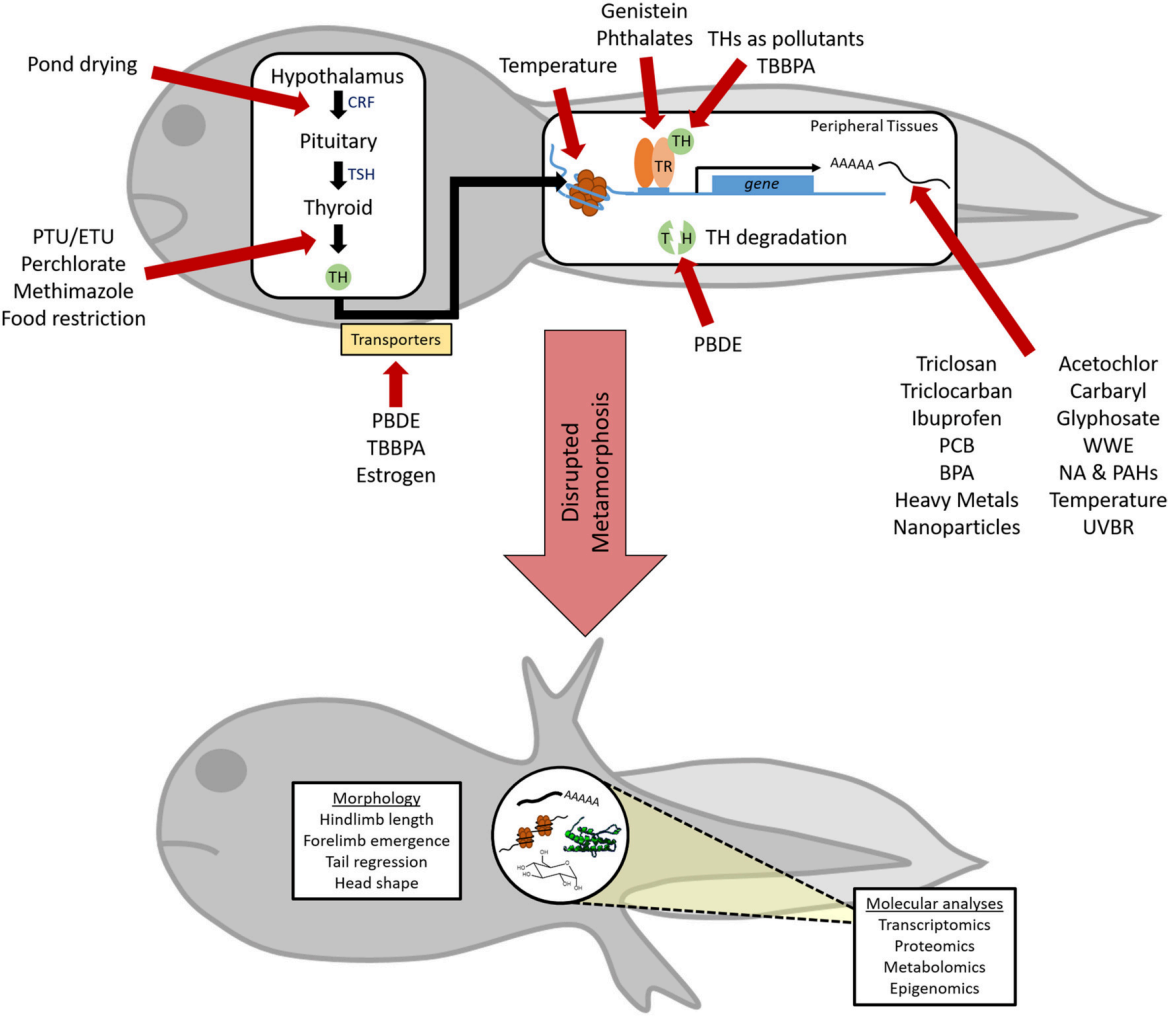
Perturbation of thyroid hormone (TH)-dependent amphibian metamorphosis by xenobiotic and abiotic exposures. Chemical and/or environmental factors can disrupt TH action at multiple points along this pathway (red arrows), although little is known about the specific mechanism of action for many factors. Due to its absolute reliance on proper TH signaling, metamorphic endpoints can be used to reveal the TH-disrupting capabilities of these factors. However, a more complete understanding of endocrine disruption and insight into modes of action can be achieved through the use of advanced techniques to assess alterations in the transcriptome, proteome, metabolome, and epigenome within metamorphosing tadpoles. CRF, corticotropin releasing factor; TSH, thyroid stimulating hormone; TR, TH receptor; PTU, propylthiouracil; ETU, Ethylenethiourea; TBBPA, Tetrabromobisphenol A; PBDE, polybrominated diphenyl ethers; PCB, polychlorinated bisphenols; BPA, bisphenol A; NA & PAHs, napthenic acid and polycyclic aromatic hydrocarbons; WWE, wastewater effluent; UVBR, ultraviolet B radiation.

In the present review, we discuss the effects of chemical and environmental disruptors of metamorphic TH signaling on anuran amphibians. Anurans are particularly tractable for the study of TH disruption due to the absolute necessity for TH to initiate metamorphosis, and consequently, the well-demarcated developmental transitions in amphibians (11). Chemical disruption of anuran metamorphosis almost exclusively originates from anthropogenic sources: industry, agriculture, pharmaceuticals, and personal care products (PPCPs; Figure 3). Additionally, environmental factors, including temperature variations and ultraviolet radiation, have demonstrated effects on metamorphosis (Figure 3). Numerous studies have examined the effects of single chemical, complex chemical mixtures, or environmental exposures on amphibian morphology during metamorphosis and we focus our discussion on those that have additionally demonstrated a TH-dependence of these effects. Adverse toxicant and environmental exposures can compromise other endocrine and molecular signaling pathways beyond TH, with sub-lethal physiological consequences for reproductive success, behavior, and broader dysfunction (16–19). We have restricted our discussion to select representatives from each of the major classes listed above and regret being unable to undertake an exhaustive review of all the excellent work done on TH disruptors.

The adoption of molecular biology techniques to assess the perturbation of TH-dependent metamorphosis has complemented conventional morphological characterizations and provided further insight into the sensitive responses of TH-induced gene expression (Figure 1) (20, 21). We discuss how the application of quantitative polymerase chain reaction (qPCR), DNA microarrays, next generation sequencing and other ‘omics techniques can ascertain TH disruption through the timely detection of biomarkers prior to the manifestation of morphological phenotypes (11, 22, 23). A list of TH-responsive gene transcripts mentioned in the current review is presented in Table 1.

**Table 1:** List of gene names utilized and their abbreviations.

**Abbreviation**	**Gene name**
*ahrl*	Aryl hydrocarbon receptor-like
*app*	Amyloid β precursor protein
*asl*	Argininosuccinate lyase
*cebp1*	CCAAT enhancer binding protein 1
*cebp2*	CCAAT enhancer binding protein 2
*cebpd*	CCAAT enhancer binding protein Δ
*cps1*	Carbamoyl phosphate synthetase 1
*crhbp*	Corticotropin-releasing factor-binding protein
*dio1*	Deiodinase 1
*dio2*	Deiodinase 2
*dio3*	Deiodinase 3
*fap*	Fibroblast activation protein α
*heket*	Heket
*hsp30*	Heat shock protein 30
*ipo*	Importin
*klf9*	Krüppel-like factor 9 formerly referred to as *bteb*
*krt1*	Cytokeratin type 1
*mbp*	Myelin basic protien
*mct8*	Monocarboxylate transporter
*mmp2*	Matrix metalloproteinase 9, formerly known as *gelatinase A*
*mmp9*	Matrix metalloproteinase 9, formerly known as *gelatinase B*
*nfic*	Nuclear factor I/C
*oatp1c1*	Solute carrier organic anion transporter family member 1c1
*otc*	Ornithine transcarbamylase
*pcna*	Proliferating cell nuclear antigen
*pparg*	Peroxisome proliferator activated receptor γ
*prlr*	Prolactin receptor
*rlk1*	Rana larval keratin I
*rxrg*	Retinoid X receptor γ
*ssh*	Sonic hedgehog
*st3*	Stromelysin 3 also known as matrix metalloprotein 11 (*mmp11*)
*thibz*	TH induced bZip protein formerly referred to as *TH/bZip, b/Zip, gene 8*, or *gene 9*
*thra*	TRα
*thrb*	TRβ
*timp2*	Tissue inhibitor of metalloproteinases 2
*trip4*	TR interactor 4
*tsha*	Thyroid stimulating hormone α
*tshb*	Thyroid stimulating hormone β
*ttr*	Transthyretin

## Pharmaceuticals and Personal Care Products

Pharmaceutical and personal care products (PPCPs) are an abundant source of diverse anthropogenic contaminants in global aquatic and terrestrial environments (24, 25). Increasing evidence links TH disruption in frogs with a variety of PPCPs, some of which are highlighted below and summarized in Table 2.

**Table 2:** Summary of PPCP effects on morphological and molecular endpoints for amphibians undergoing both natural and TH-induced metamorphosis.

			**Metamorphosis**		**Morphological/Behavioral**	**Molecular**			
***Category***	**Chemical**	**Species**	**Natural**	**Induced**	**Results**	**Tissue**	**Technique**	**Result**	**References**
Antimicrobial	Methyl Triclosan	*R. catesbeiana*	Y			C-fin^d^	qPCR	*↑rlk1, thrb*	(26)
	Triclocarban	*R. catesbeiana*	Y			C-fin	qPCR	*↓rlk1*	(26)
	Triclosan	*Pseudacris regilla*		T_4_	↑ metamorphic rate	Brain	qPCR	*↑pcna, thra, thrb*	(27)
				T_4_		Tail	qPCR	*↑thra; ↓thrb*	(27)
		*R. catesbeiana*		T_3_	↑ metamorphic rate	Brain	qPCR	*↑pcna, thrb*	(28)
				T_3_		C-fin	qPCR	no effect on *hsp30, rlk1, thrb*	(28)
				T_3_		Tail	qPCR	*↓thrb*	(28)
		*X. laevis*		T_3_		XTC-2 cell line	qPCR	*↑klf9, thra, thrb*	(28)
Estrogen	E_2_^a^	*R. catesbeiana*	Y			Olfactory epithelium	qPCR	*↑st3, thibz*	(6)
	E_2_^b^	*R. catesbeiana*	Y			Olfactory epithelium	RNA-seq (52,699 contigs)	Δ 267: 112 overlap with TH	(6)
Pharmaceutical	Ibuprofen	*R. catesbeiana*	Y	T_3_		C-fin	qNPA^c^,qPCR	*↑ahrl, cebp2, dio3; ↓prlr*	(29)
				T_3_		Liver	qNPA,qPCR	*↑asl, thra, thrb*	(29)
				T_3_		Liver	cDNA Array (MAGEX; 434 genes)	Δ 27: transcription, calcium transport, proteolysis, cell cycle, protein phosphorylation	(29)
			Y			Liver	cDNA Array (MAGEX; 434 genes)	Δ 26: oxygen transport, arginine metabolism, urea production	(29)
	Methimazole	*X. laevis*	Y		↓ metamorphic rate	Brain	qPCR	*↓app, thra*	(30–33)
			Y			Brain	cDNA Array (MAGEX; 434 genes)	[24 h] ↑4↓35: chromatin structure, signal transduction, transcription; [48 h] ↑12↓7: cell growth control, chromatin structure, structural, signal transduction, transcription; [96 h] ↑4↓30: apoptosis/protein processing, cell growth control, chromatin structure, hormonal regulation, metabolism, signal transduction, structural, transcription, translation, transport/binding	(32)
			Y			Hindlimb	qPCR	*↑ipo, krt1; ↓thra*	(34)
			Y			Hindlimb	cDNA Array (MAGEX; 434 genes)	↑11: cell growth control, hormonal regulation, protein processing, signal transduction, structural, transcription, transport/binding	(34)
			Y			Tail	qPCR	*↓ipo*	(34)
			Y			Tail	cDNA Array (MAGEX; 434 genes)	↑4↓1: hormonal regulation, structural, protein processing, signal transduction	(34)
	Propylthiouracil	*X. laevis*	Y		↓ metamorphic rate	Brain	qPCR	*↑mbp, ↑plp*	(31, 32)
			Y			Brain	cDNA Array (MAGEX; 434 genes)	[24 h] ↑3↓36: transcription; [48 h] ↑8↓11; [96 h] ↑9↓29: transcription, hormonal regulation, structural	(31, 32)
			Y			Tail	qPCR	*↑krt1*	(31, 34)
			Y			Tail	cDNA Array (MAGEX; 434 genes)	[48 h] Δ 4: transcription, cell growth control, transport/binding	(31, 34)
			Y			Hindlimb	qPCR	*↑krt1*	(31, 34)
			Y			Hindlimb	cDNA Array (MAGEX; 434 genes)	[24 h] Δ 7: hormonal regulation, structural; [96 h] Δ 7: protein processing, transcription, transport/binding, signal transduction	(31, 34)
	Ethylenethiourea	*X. laevis*	Y		↓ metamorphic rate	Brain	qPCR	*↑dapl1; ↓thrb, pcna, mcm2, kif2C*	(35)
			Y			Pituitary	qPCR	*↑tshb*	(35)
			Y			Thyroid tissue	qPCR (60 gene screen)	↑43↓6: TH synthesis, secretion, metabolism; protein synthesis and transport, growth arrest, apoptosis, cellular stress responses	(35)
Thyroid hormone	T_3_^b^	*R. catesbeiana*		T_3_		Olfactory epithelium	RNA-seq (52,699 contigs)	Δ 38,830: sensory perception, DNA repair, mitochondrial energetics, transcription and RNA processing, endoplasmic reticulum	(6)
	T_3_^a^	*R. catesbeiana*		T_3_		Back skin	qPCR	*↑cebp1, klf9, thibz; ↓rlk1*	(7)
				T_3_		Brain	qPCR	*↑dio2, klf9; ↓rlk1*	(7)
				T_3_		Intestine	qPCR	*↓klf9, rlk1, thrb*	(7)
				T_3_		Liver	qPCR	*↑cebp1*	(7)
				T_3_	↓ predator cue avoidance	Olfactory epithelium	qPCR	*↑dio2, heket, st3, thibz, thra, thrb*	(6, 36)
				T_3_		Tail fin	qPCR	*↑thibz*	(7)
	T_4_^b^	*R. catesbeiana*		T_4_		Olfactory epithelium	RNA-seq (52,699 contigs)	Δ 31,439: sensory perception, potassium ion transport, DNA repair, mitochondrial energetics, transcription and RNA processing	(6)
	T_4_^a^	*R. catesbeiana*		T_4_		Back skin	qPCR	*↑cebp1, klf9, thibz*	(7)
				T_4_		Brain	qPCR	*↑dio2*	(7)
				T_4_		Intestine	qPCR	*↑thra*	(7)
				T_4_		Liver	qPCR	*↑thrb*	(7)
				T_4_		Olfactory epithelium	qPCR	*↑dio2, heket, st3, thibz, thra, thrb, trpv1*	(6)

a *Contaminant at environmental levels where physiological level data was collected*.

b *Contaminant at physiological levels*.

c *Quantitative nuclease protection assay*.

d *Cultured tail fin assay*.

### THs as Pollutants (T_3_/T_4_)

THs can be found as pollutants in environmental water systems. As thyroid medication is the third-most prescribed drug in Canada for women aged 25–66, TH can be found in municipal wastewater (37). Brown and Wong measured the concentrations of T_4_ at a wastewater treatment plant in Winnipeg, Canada and found a range from 60 to 79 ng/L (~0.1 nM) with T_4_ persisting through the treatment phases (38). The majority of recent studies examining precocious metamorphosis induced by THs have used physiological levels (e.g., 10–50 nM). More recently, however, studies have shown that premetamorphic tadpoles are competent to respond to lower, more environmentally-relevant levels of T_3_ and T_4_ found in wastewater (6, 7). Maher et al. found that in *Rana* [*Lithobates*] (*R*.) *catesbeiana dio2* and *cebp1* are responsive to as little as 0.05 nM T_4_ in the brain and back skin, respectively (7). Slightly higher concentrations of 0.1 nM T_3_ and 0.5 nM T_4_ led to an increased number of TH-responsive transcripts such as *thrb, thibz, klf9*, and *rlk1* in the back skin, brain, intestine, liver, and tail fin (Table 2). In the same species, Jackman et al. found that olfactory epithelium exposed to 0.5 nM T_4_ also exhibited a significant increase in *thrb, thra, and thibz* (7). The responsiveness of TH-linked transcripts to environmentally-relevant levels of THs indicates that these low concentrations may be enough to affect metamorphosis. An early study demonstrating TH-induced metamorphosis found that premature induction resulted in mortality when TH amounts were greater than environmental levels (7).

Exposure to T_3_ is also associated with behavioral changes in which tadpoles lose the ability to detect a predator cue (36). Surprisingly, comparable T_4_ exposures had no effect on this behavioral endpoint (36). Molecular analyses of the olfactory epithelium using qPCR and RNA-seq methods revealed that this tissue was extraordinarily sensitive to both hormones and, while many gene responses were shared between the two hormones, a substantial number were unique to each hormone with T_3_ significantly affecting a 14 more contigs than T_4_ (6, 7). Notable differences in sensory perception, potassium ion transport, DNA repair, mitochondrial energetics and transcription/RNA processing gene ontologies provide some insight into the different effects of these hormones (36). These studies accentuate that the two TH contaminants should be treated separately when looking at responses to environmentally-relevant levels of THs.

### Propylthiouracil and Ethylenethiourea

6-Propylthiouracil (PTU) is a TH synthesis antagonist that is clinically used to treat hyperthyroidism. Ethylenethiourea (ETU) is also an anti-thyroidal compound that, similar to PTU, inhibits thyroid peroxidase, the enzyme that synthesizes TH (39). *Xenopus* (*X*.) *laevis* tadpoles independently exposed to PTU and ETU had inhibited metamorphic progression (30, 40). *X. laevis* tadpoles exposed to ETU at stage 51 exhibited delays and arrest of natural metamorphosis, as measured by forelimb emergence (21). Histological aberrations in thyroid gland formation were evident with increased glandular size and follicle size and partial colloid depletion following exposures to ETU and PTU (21, 30). Elevated abundance of *tsha* and *tshb* transcripts were measured by qPCR in the pituitary tissue of tadpoles exposed to ETU (21). Similar metamorphic delays and aberrant thyroid gland histology were also observed in *X*. (*Silurana*) *tropicalis* and *R. rugosa* tadpoles following PTU exposures (41, 42).

Early prometamorphic *X. laevis* tadpoles (Niewkoop and Faber [NF] stage 54) exposed to 20 mg/L PTU did not have significantly altered *thra, thrb*, or *klf9* transcript abundance in the brain, hindlimb or tail (31, 43). MAGEX cDNA array analysis of naturally metamorphosing *X. laevis* tadpoles at NF stage 54 exposed to PTU recorded a greater number of transcripts with decreased abundance than increased abundance in the brain at 24, 48, and 96 h post-treatment (Table 2) (32). Differential transcription was ontologically associated with transcriptional regulation at 24 h and at 96 h, transcription, hormonal regulation and structural proteins (32). Correspondence analysis was used to identify possible metamorphic biomarker candidates and qPCR analyses confirmed the increased expression of myelin basic protein (*mbp*) and myelin proteolipid protein (*plp*) in the brain upon PTU exposure (Table 2) (32). Using similar experimental conditions, the PTU-dependent effects were further examined in the *X. laevis* hindlimb and tail (34). Seven transcripts were identified by cDNA arrays to have differential abundance in the hindlimb at 24 and 96 h post-exposure and were associated with hormonal regulation and structural proteins at 24 h and protein processing, transcription, and transport and binding at 96 h (Table 2) (34). Using cDNA arrays, 4 transcripts were detected to have differential levels in the tail at 48 h and were linked to transcription, cell growth control, and transport and binding ontologies (Table 2) (34). Potential biomarkers were screened using qPCR and cytokeratin type I (*krt1*) transcripts were elevated significantly in both the hindlimb and tail (Table 2) (34).

Naturally metamorphosing *X. laevis* tadpoles exposed to ETU exhibited developmental arrest and aberrant thyroid histology: goiter formation, colloid depletion and follicular cell hypertrophy and hyperplasia (35). Treatment with this goitrogen induced significant decreases in *thrb, klf9, pcna, mcm2, kif2C*, and increased *dapl1* transcript abundance in the brain as measured by qPCR (35). ETU treatment also resulted in increased *tshb* transcripts in the pituitaries. A qPCR candidate biomarker screening was performed on thyroid tissue and 49 of 60 genes had significantly differential abundance following ETU exposure compared to the controls (35). Of these, 43 genes had increased transcript abundance, while six were decreased. These ETU-induced differential transcripts were ontologically associated with the synthesis, secretion, and metabolism of THs, protein synthesis and transport, growth arrest, apoptosis, and cellular stress responses (35).

### Methimazole

Methimazole is an established disruptor of amphibian HPT axis function and has been frequently used as a metamorphosis inhibitor (30). Similar to PTU and ETU, methimazole is a goitrogen and anti-thyroid drug that affects TH signaling by inhibiting thyroid peroxidase (44). Exposure to methimazole for 14 days during metamorphosis resulted in a significantly decreased metamorphic rate in pre- and prometamorphic *X. laevis* tadpoles and thyroid gland hypertrophy and follicular cell hyperplasia (Table 2) (30, 33). The molecular effects of up to 72 h of methimazole exposure on early prometamorphic *X. laevis* tadpoles were queried by qPCR analysis of known TH-regulated genes. Zhang et al. found a significant decrease in *thra* and *app* gene expression in the brain; a decrease in *thra* and increase in *ipo* and *krt1* mRNAs in the hindlimb; and a decrease in *ipo* transcripts in the tail (Table 2) (36, 38, 39). Helbing et al. used cDNA arrays to further evaluate the pathways affected by methimazole in *X. laevis* tadpoles (Table 2) (32, 34). In the brain, an increase of 20 and decrease of 76 gene transcripts related to transcription, hormonal regulation, and structural pathways was observed (32). In the hindlimb, the 11 increased transcripts were related to cell growth control, hormonal regulation, protein processing, signal transduction, structural, transcription, and transport/binding pathways. The tail had four increased and one decreased transcript that were related to hormonal regulation, structural, protein processing and signal transduction pathways (34). Ontological analyses of differentially affected brain transcripts were associated with apoptosis/protein processing, cell growth control, chromatin structure, hormonal regulation, metabolism, signal transduction, structural, transcription, translation, and transport/binding pathways with qPCR analysis revealing an increase in *ipo* and *krt1* and a decrease in *thra* mRNA levels (Table 2) (34).

### Estrogen

The steroid hormone and TH axes are closely related. As the synthesis of both endocrine hormones is controlled through hypothalamic-pituitary axes and both bind nuclear receptors that stimulate gene expression cascades, it is unsurprising that there is some cross-talk between these two pathways. The majority of studies that have looked at the effects of 17β-estradiol (E_2_) or the synthetic estrogen 17α-ethinylestradiol (EE_2_) on metamorphosis have found a decreased metamorphic rate (Supplementary Table 1) (32–35, 44) [reviewed by Hayes (45)]. However, Frieden & Naile found accelerated tail reduction in *Bufo* (*B*.) *bufo* upon exposure to estrone (E_1_) (46). How estrogens influence TH signaling is not completely understood. In adult *R. ridibunda*, E_2_ decreases plasma T_3_ and T_4_ (47), although this may not occur during metamorphosis. Brande-Lavridsen et al. found that during metamorphosis in *R. temporaria*, there was no significant difference in total or free T_3_ upon exposure (48). However, Yamauchi et al. found that both E_2_ and the synthetic estrogen diethylstilbestrol could competitively bind with recombinant *X. laevis* and *R. catesbeiana* transthyretins; TH transporter proteins (49) (Supplementary Table 2). The thyroid itself was found to show no change in number of follicles or overall thyroid volume, although there was a decreased follicular cell height upon exposure to EE_2_.

To determine the response of the gene program, Jackman et al. investigated the transcriptomic effects of E_2_ in the olfactory epithelium of *R. catesbeiana* and found none of the classic TH-response genes, such as *thra, thrb, thibz*, or *dio2* changed upon an acute exposure to either environmentally-relevant or higher levels of E_2_ (6). This is corroborated by Bulaeva et al. who exposed *R. sylvatica* to much higher levels of E_2_ and still saw no significant response of *thrb* (50). With more in-depth RNA-seq analysis, Jackman et al. found 112 significantly changing contigs that also responded to exposure to T_3_ and/or T_4_ (6). However, compared to almost 45,000 contigs that respond to exposure to TH, this cross-talk signaling is quite minimal. As estrogens are found throughout our wastewater systems (51), it is imperative to determine the mechanism by which estrogens are affecting with TH signaling and proper development.

### Triclosan and Triclocarban

Triclosan [5-chloro-2-(2,4-dichlorophenoxy)phenol; TCS] is a bactericidal and antifungal agent that is ubiquitously incorporated into thousands of industrial and consumer products including clothing, toys, cleaning supplies, personal care products (i.e., soap, shampoo, toothpaste, etc.), and surgical soaps and sutures (52, 53) with 10.5 million pounds produced globally in 2015 (53). Triclosan and triclocarban (TCC), another widely used antibacterial in PPCPs, are the most common, broad-spectrum antimicrobial agents used in household items and PPCPs (54). While sewage treatment removes most triclosan, it still contaminates sewage effluent and, consequently, aquatic environments (24). The U.S. Food and Drug Administration banned the use of TCS, TCC, and 17 other antimicrobials in personal wash products in 2016 to minimize the exacerbation of bacterial resistance and health risks, including endocrine disruption (54, 55). TCS has structural similarity to TH and disruption of TH action in frogs provided some of the earliest evidence of this endocrine disruption.

Low and environmentally-relevant amounts of TCS can affect different aspects of TH signaling in amphibians (30, 41, 51–57). Exposure of premetamorphic *R. catesbeiana* tadpoles to environmentally-relevant amounts of triclosan can induce altered growth and transcript responses that are exacerbated upon T_3_-induced metamorphosis (28). The combinatorial effects of TCS and T_3_ on tadpoles resulted in greater body mass reductions and precocious metamorphosis. These phenotypic changes were accompanied and preceded by changes to TH-responsive gene expression (28). Expression of *thrb* was transiently decreased in the tadpole tail at 48 h, while the brain had increased expression of *thrb* and proliferating cell nuclear antigen transcripts (*PCNA*). Under comparable TCS ± T_3_ treatments, cultured *X. laevis* XTC-2 cells had increased expression of *thra, thrb, and klf9* after exposure to both chemicals, supporting the developmentally-sensitive TCS effects in different anuran species (28). Recent work demonstrated that *X. tropicalis* exposed to TCS levels considered safe in drinking water developed metabolic pathologies resembling prediabetes and produced progeny exhibiting delayed metamorphosis and diminished reproductive success (58).

Adaptation of the Amphibian Metamorphosis Assay for the Pacific tree frog, *Pseudacris* (*P*.) *regilla*, (TREEMA) revealed comparable morphological and molecular disruption by TCS when administered in conjunction with T_4_ (27). By the second day of exposure, TCS enhanced the T_4_-stimulated increases in *thra, thrb, and pcna* in the tadpole brain and disrupted expression of TH-responsive genes in the tail (Table 2) (27). The earliest morphological effects of TCS and T_4_ exposures were evident at day 4 with increased foot paddle formation and later impairments in developmental stage progression. Tadpoles exposed to both TCS and T_4_ also had accelerated development and increased hindlimb length/snout-vent length ratio (27). Like other anurans, the perturbed metamorphic profile in *P. regilla* is indicative of disrupted developmental coordination (27). Exposure of *X. laevis* tadpoles to TCS resulted in increased *thrb* mRNA in the tail fin after 21 days followed by thyroid gland hypertrophy at 32 days (Table 2) (56, 57, 59, 60).

Methyl triclosan (mTCS) is a bacterial metabolite of TCS and is more persistent in the environment than TCS, which is readily degraded by photolysis (61). This metabolite, along with TCS and TCC, were tested using premetamorphic *R. catesbeiana* cultured tail fin (C-fin) assays. TCS did not affect TH-responsive *rlk1* or *thrb* transcript abundance, but did increase *hsp30* levels (Table 2) (26). mTCS exposure increased both *rlk1* and *thrb* transcripts in the absence of T_3_ (26), suggesting that some, but not all, of the TCS activity observed in intact animals may be due to the conversion to mTCS. TCC exposure caused a reduction in *rlk1* transcripts and an increase in *hsp30* mRNA (Table 2) (26), indicating a TH-like activity of this antimicrobial agent.

### Ibuprofen

Ibuprofen is a commonly used non-steroidal anti-inflammatory analgesic that is now a prevalent component of complex municipal wastewater effluents that permeate aquatic environments (62, 63). Ibuprofen is primarily considered to act through prostaglandin synthesis inhibition, however, it can also interfere with multiple regulatory pathways (29, 64). Little is known about the effects ibuprofen can have on aquatic organisms during sensitive developmental periods, which is concerning given the multiplicity of molecular pathways ibuprofen targets and its abundance in global freshwater environments.

Exposure of *R. catesbeiana* tadpoles to environmentally-relevant concentrations of ibuprofen disrupted TH-stimulated metamorphic reprogramming of the liver transcriptome and in C-fin assays (Table 2) (29). MAGEX cDNA microarray analyses of tadpole livers exposed to ibuprofen and T_3_ detailed molecular pathways affected by these combined exposures: transcription, calcium transport, proteolysis, cell cycle, and protein phosphorylation. Additionally, ibuprofen treatment affected pathways related to oxygen transport, arginine metabolism and urea production (29). Ibuprofen exposure of T_3_-stimulated tadpoles enhanced the upregulated expression of *thra* and *thrb*. Quantitative nuclease protection assay analysis of C-fin cultures showed that ibuprofen exposure alone could increase expression of *dio3*, while both ibuprofen and T_3_ treatment resulted in an increase in *hsp30* transcripts, indicating potential tissue-specific responses (29). Ibuprofen can also affect transcriptional programs in the tail fin and back skin of *R. catesbeiana* under temperature-dependent, T_3_-stimulated conditions and this is further discussed below (65).

## Industrial and Agricultural Chemicals

### Polychlorinated Bisphenols (PCBs)

Polychlorinated bisphenols (PCBs) are ubiquitous environmental contaminants that were widely used in capacitors and transformers between 1929 and 1979 (66). Concern about the endocrine disrupting potential of PCBs resulted in their import and use being banned in North America by 1979. However, the extreme environmental persistence and bioaccumulation of PCBs continue to plague us (66). With the effects of PCBs on TH homeostasis well-characterized (67), there was a clear need to investigate the effect of these compounds on amphibian metamorphosis.

As the toxicity of PCBs is typically due to bioaccumulation over time, Gutleb et al. examined the effects of ingested PCBs in *R. temporaria* and *X. laevis* after an exposure of either 10 days or several weeks (68). They found that dietary exposure to a technical mixture of PCBs, clophen A50, decreased metamorphic rate in both species after 10 days. Furthermore, exposures to PCB 126 decreased the rate of metamorphosis after several weeks (Supplementary Table 1). In a later study, Gutleb et al. showed that immersion in PCB 77 and apolar sediment extracted from PCB-contaminated ponds significantly reduced the rate of metamorphosis in *X. laevis* (Supplementary Table 1) (69). Gutleb et al. confirmed these effects using a *X. laevis* thiourea-synchronized metamorphosis assay and a 60 day dietary exposure. In this study, they found that clophen A50 and an apolar sediment extract from polluted ponds decreased the rate of metamorphosis (Supplementary Table 1) (70).

To assess the effects of PCB exposure on TH-mediated gene expression, Lehigh et al. examined the toxicity of another technical mixture of PCBs, A1254 (71). qPCR analysis of pooled mRNA from *X. laevis* tadpoles showed that A1254 exposures decreased *dio2* and *dio3* expression and increased *ttr* expression (Table 3). These results, in combination with the previous studies performed by Gutleb et al., show that mixtures of PCBs exhibit significant effects on TH-driven amphibian metamorphosis (Table 3).

**Table 3:** Summary of industrial and agricultural chemical effects on morphological and molecular endpoints for amphibians undergoing both natural and TH-induced metamorphosis.

			**Metamorphosis**		**Morphological**	**Molecular**			**References**
***Category***	**Chemical**	**Species**	**Natural**	**Induced**	**Result**	**Tissue**	**Technique**	**Result**	
Flame retardants	A1254 (technical PCB mixture)	*X. laevis*	Y		↓ metamorphic rate	Whole tadpole	qPCR	*↑ttr; ↓dio2, dio3*	(71)
	BDE-47	*X. laevis*	Y		↓ metamorphic rate	Brain	qPCR	*↓dio2, klf9, mct8, oatp1c1, thra, thrb, tshb*	(72, 73)
	TBBPA	*Pelophylax nigromaculata*	Y			Intestine	qPCR	*↑mmp2, thibz*	(74)
				T_3_		Intestine	qPCR	*↓klf9, mmp2, ssh, thibz, thrb*	(74)
		*Pseudacris regilla*		T_3_	↑ metamorphic rate	Brain	qPCR	*↑thra*	(75)
				T_3_		Tail	qPCR	*↑mmp9; ↓pcna*	(75)
		*X. laevis*		T_3_	↓ metamorphic rate	Head	qPCR	*↓thibz, thrb*	(76–80)
			Y			HeLa cells	GAL4-luciferase reporter assay with *Xenopus* TRα ligand binding domain	↓activation	(77)
				T_3_		Hindlimb	qPCR	*↓dio2, st3, thrb*	(80)
				T_3_		Intestine	qPCR	*↓dio2, klf9, mmp2, thrb*	(80)
				T_3_		Tail	qPCR	*↓cebpd, dio3, klf9, st3, thrb*	(79)
				T_3_		Transgenic tadpoles	*thibz*-luciferase reporter	↓activation	(78)
				T_3_		Transgenic tadpoles	*thibz*-GFP reporter	↓activation	(77)
Isoflavonoid	Genistein	*R. catesbeiana*	Y			C-fin	qPCR	*↓thrb*	(81)
				T_3_		C-fin	qPCR	*↓thrb*	(81)
				T_3_	↓ tail regression	Tail tip culture	qPCR	*↓thrb*	(82)
				T_3_		Tail tip culture	Western blot	↓tyrosine phosphorylation	(82)
				T_3_		Tail tip culture	Western blot	↓activity of protein kinase C	(82)
				T_3_		Tail tip culture	Western blot	↓TRα phosphorylation levels	(82)
				T_3_		Tail tip culture	Western blot	↑TRα	(82)
Metals	Cd	*B. gargarizans*	Y		↓ metamorphic rate	Whole tadpole	qPCR	[5 μg/L] ↑*thra*; [50 μg/L] ↓*thra*; [100 μg/L] ↓*dio2 thrb*	(83)
	Cd/Te quantum dots	*R. catesbeiana*		T_3_		C-fin	qPCR	*↓rlk1, thrb*	(84)
	Cu	*B. gargarizans*	Y		↓ metamorphic rate	Whole tadpole	qPCR	*↑dio3; ↓dio2, thra, thrb*	(85)
	Hg	*B. gargarizans*	Y		↓ metamorphic rate	Liver	qPCR	*↓dio2, thra, thrb*	(86)
	Nanosilver	*R. catesbeiana*		T_3_		C-fin	qPCR	*↓rlk1, thrb*	(84)
	Nanosilver	*X. laevis*	Y			Liver	cDNA Array (MAGEX, 497 genes)	[Premet^a^] ↑3↓4: Myotube cell development, Protein binding, Proteolysis, Oxidative stress, ATP biosynthesis, Transcriptional regulation, Cell cycle arrest; [Promet^b^] ↑12↓4: Regulation of transcription, Nervous system development	(87)
Pesticide	Acetochlor	*R. catesbeiana*	Y			Brain	qPCR	*↑thra, thrb*	(88)
				T_3_		C-fin	qPCR	*↑thrb*	(89)
				T_3_		Tail	qPCR	*↑thra, thrb*	(88)
		*X. laevis*		T_3_	↑ metamorphic rate	Tail	qPCR	*↑thra, thrb*	(90)
				T_3_		Tail	cDNA Array (MAGEX, 420 genes)	Altered pathways: transcription factors, apoptotic proteins, signaling molecules, enhanced expression of T_3_-responsive genes	(90)
	Carbaryl	*R. clamitans*	Y			Brain	qPCR	*↑thra, thrb*	(91)
			Y			Brain	cDNA Array (MAGEX, 420 genes)	Altered pathways: transcription, cell growth control, signal transduction	(91)
			Y			Tail	qPCR	*↑thra*	(91)
	Roundup Original and Transorb	*R. pipiens*	Y		↑ metamorphic rate	Tail	qPCR	*↑thrb* (premets, not metamorphic climax)	(92)
Plastics Additive	BPA	*X. laevis*	Y	T_3_	↓ tail regression	Cultured tail	PCR	*↓thrb*	(93)
				T_3_	↓ metamorphic rate	Intestine	qPCR	*↓mmp2, st3, thibz, timp2*	(94)
				T_3_		Intestine	DNA microarray (Affymetrix)	↓T_3_ signaling pathways	(94)
	Dicyclohexyl phthalate	*X. laevis*		T_3_		XL58 cell line	*thibz*-luciferase reporter; qPCR	↓activation; ↓*thrb*	(95)
	Di-n-butyl phthalate	*X. laevis*		T_3_	↓ metamorphic rate	Head		↑*tsh*α, tshβ; ↓*thrb*, RXRγ	(96)
				T_3_		XL58 cell line	*thibz*-luciferase reporter; qPCR	↓activation; ↓*thrb*	(95)
	Mono-n-butyl phthalate	*X. laevis*		T_3_	↓ metamorphic rate	Head		↑*tsha, tshb*; ↓*rxrg, thrb*	(96)
						Head	Bisulfite sequencing	↓methylation in *thrb* promoter	(96)
	N-butylbenzyl phthalate	*X. laevis*		T_3_		Whole tadpole		↓*thrb*	(95)
				T_3_		XL58 cell line	*thibz*-luciferase reporter; qPCR	↓activation; ↓*thrb*	(95)

a *Premetamorphic tadpoles*.

b *Prometamorphic tadpoles*.

### Perchlorate

Perchlorates, such as ammonium perchlorate, potassium perchlorate, and sodium perchlorate, are well-known as powerful oxidizing agents, which has led to their widespread usage in explosives such as rocket propellants, fireworks, and signal flares (97). They are also used to treat TH diseases (98) as perchlorates competitively inhibit the uptake of iodine by the sodium-iodide symporter, leading to lack of iodine for the production of THs (99). Unfortunately, due to its widespread industrial use, perchlorate is a persistent pollutant. As amphibians have an almost identical TH system to humans, it is unsurprising that perchlorates also affect their TH-regulated processes [reviewed by Carr and Theodrakis (100)], leading to a decreased metamorphic rate (31, 35, 50, 101–103). Chronic exposures to environmental levels of perchlorate decrease T_4_ in *X. laevis*, both *in vivo* (104) and *in vitro* (105). This indirectly results in the enlargement of the thyroid glands as well as hyperplasia and hypertrophy of thyroid follicles due to the lack of negative regulation of TSH (20, 35, 104, 106, 107). Predictably, the decrease in T_4_ levels also leads to decreased metamorphic rate*s* (35, 101, 102, 106, 107).

The involvement of the TH-induced gene expression program in this metamorphic delay seems to be organ-dependent. Using cDNA array analyses of acute exposures of sodium perchlorate in *X. laevis*, Helbing et al. found that the brain was the most responsive with a maximum of 39 responsive genes involved mostly in transcription, transport/binding, apoptosis/protein processing, and structure (Table 3) (32). *Tshb* mRNA significantly increased after 48 h, suggesting an acute exposure already leads to dysregulation of the negative feedback loop. The cDNA array only indicated 8 and 4 responsive genes in the tail and hindlimb, respectively (34), indicating that these tissues may be less responsive to acute exposures of perchlorate. However, in chronic exposures of environmentally-relevant levels of perchlorate, there is a more consistent response. Flood & Langlois (108) observed decreased TH-responsive genes, *thra*, and *thrb*, in the liver of *X. tropicalis* chronically exposed to potassium perchlorate. A similar result was seen in the brain of *X. laevis* chronically exposed to sodium perchlorate (Table 3) (35). Bulaeva et al. (50) found that *R. sylvatica* had decreased *thrb* transcript levels in the tail and liver, which could be continually observed even 40 days after a 2 week exposure to sodium perchlorate, indicating that the effects from perchlorate may be persistent and possibly irreversible.

### Brominated Flame Retardants (BFRs)

Brominated flame retardants (BFRs) have been and continue to be ubiquitously incorporated into a variety of items to confer fire resistance (109). These materials include textiles, plastics, electronic circuitry, wood, paper, dust, and inadvertently in the 1970's, livestock feed (109–111). Roughly 5,000,000 metric tons of bromine are produced worldwide annually, with demand increasing each successive year (111). BFRs include polybrominated diphenyl ethers (PBDEs), polybrominated biphenyls (PBBs), tetrabromobisphenol A (TBBPA) and hexabromocyclododecane (HBCD). Depending upon the mechanism by which BFRs are integrated within materials, BFRs can be classified as brominated monomers, reactive (i.e., TBBPA) or additive (i.e., PBDE, HBCD). BFRs can readily leach from materials if they are not strongly chemically bound to the composite polymer, thereby contaminating the environmental biota, leading to mortality, compromised development and other toxicity-dependent pathologies among animal populations. A growing concern is that increasing amounts of BFRs have been found in the environment throughout different trophic levels, including humans, underscoring the need to better understand the biological implications of BFRs (111). Many BFRs are lipophilic and this facilitates their persistent bioaccumulation in the biota of both aquatic and terrestrial environments (112). Due to the deleterious effects of penta- and octa-BDE BFRs and PBBs, they have since been banned, which has spurred the development of novel BFRs (111). However, the environmental effects of these novel BFRs, which are not limited to TBBPA derivatives, are under increasing scrutiny (110, 113). Herein, we review BFRs that have a demonstrated effect on amphibian metamorphosis (Table 3).

#### Polybrominated Diphenyl Ethers (PBDE)

PBDEs are widely disseminated throughout invertebrates, vertebrates, sediments, and diverse environments, including Arctic marine biota (72). PBDEs can readily accumulate and magnify within trophic levels (114). Mammalian biotransformation of PBDEs to hydroxylated metabolites by cytochrome P450 enzymes result in products that are more toxic than the parent congeners. As previously reviewed, these metabolites can disrupt thyroid homeostasis via several mechanisms including: decreased free and total TH through the competitive binding of thyroid transport proteins and perturbed TH metabolism through glucuronidation, sulfation, and deiodination (72). Notably, there are strong structural similarities between THs and PBDEs.

*X. laevis* tadpoles (NF stage 50) fed 1,000 or 5,000 μg/g of a commercial mix of PBDE congeners, DE-71, exhibited significant inhibition of metamorphosis as displayed by delayed limb development and tail resorption, lack of pigmentation and head shape changes (72). No major cellular or morphological differences of the thyroid gland were observed following histological analyses. Intraperitoneal injections of DE-71 and BDE-47, but not BDE-99, resulted in delayed metamorphosis through significant reductions in tail resorption (72). Both BDE-47 and BDE-99 are major congeners of DE-71. Although the morphological results of this study implied the disruption of TH activity, such involvement could not be conclusively ascertained.

*R. pipiens* tadpoles fed lower, environmentally relevant amounts of DE-71 at Gosner stage 25 to stage 42 had delayed metamorphic climax by 22–36 days (3, 115). The elimination of PDBEs following depuration was studied in *R. pipiens* tadpoles that had consumed environmentally-relevant concentrations of DE-71 for 50 days at Gosner stage 25. Following 28 days of depuration, tadpoles had removed more than 94% of PBDE congeners from their bodies (114). The ability to eliminate PBDEs from tissues can vary according to life stage. Metamorphosing frogs (Gosner stage 42–46) were unable to eliminate PBDEs following depuration, however, juvenile frogs eliminated 89.7% of PBDEs over a 70 day depuration (114). Wild *R. limnocharis* adult frogs found proximal to contaminated e-waste recycling sites similarly showed reduced PBDE levels following 54 days of depuration (116).

A link between PDBE-altered amphibian metamorphic morphology and disrupted TH metabolism was demonstrated by the treatment of *X. laevis* tadpoles with increasing concentrations of BDE-47 (73). After a 21 day BDE-47 dietary exposure, tadpoles exhibited reduced developmental stage progression and decreased hindlimb length. Histological analysis of the thyroid gland showed decreased follicular epithelial cell height and a smaller thyroid lobe area in tadpoles exposed to BDE-47 (73). Corresponding reductions in hindlimb length were observed in *X. tropicalis* tadpoles following BDE-47 exposure (117). BDE-99 exposure in *X. tropicalis* similarly resulted in slower developmental stage progression and reduced hindlimb length (117). qPCR analyses in *X. laevis* to assess transcriptomic changes in the tails and livers of stage-matched tadpoles between NF stage 52 to 56 found tissue-specific TH-dependent regulation (73). No significant differences were observed in tail *thra, thrb, dio1*, or *dio2* transcripts. However, the brain was sensitive to BDE-47 treatment and significant reductions were observed in *thra, thrb, klf9, tshb, dio2, mct8*, and *oatp1c1* mRNA (73). The diversity of affected transcripts underscores the broad extent to which thyroid metabolism is adversely affected by BDE-47.

#### Tetrabromobisphenol A (TBBPA)

Tetrabromobisphenol A (2,2′,6,6′-tetrabromo-4,4′-isopropylidenediphenol; TBBPA) is one of the most abundantly used BFRs, with 150,000 metric tons produced each year. Although the majority of TBBPA is covalently bound within polymer materials, ~10–20% can leach into the proximal environment (118, 119). As such, TBBPA is found dispersed within environments around the world and in the tissues of affected organisms (112, 119). TBBPA was introduced as a replacement for PBDEs, in part due to their comparatively short half-life in mammals (120). However, TBBPA has been detected in environmental samples and humans, including breast milk (121). TBBPA bears structural similarity to T_4_ and binds to human transthyretin more strongly than T_4_ (122), but is weak competitor to T_3_ for binding TRα in rat (123). TBBPA is also reported to disrupt T_3_ binding to TRs in rat (123).

TBBPA antagonizes tail resorption during TH-mediated metamorphosis in the wrinkled frog, *R. rugosa*, and the T_3_-associated gene expression of *thrb* and *thibz* in *X. laevis* (76, 123). TBBPA can also act as a TH agonist during metamorphosis in *P. regilla* (75). These contradictory findings may reflect unique endocrine sensitivities due to differential anuran metamorphic trajectories (124). *P. regilla* tadpoles (Gosner stages 30–31) exposed to 10 nM TBBPA had increased tail regression and *mmp9* expression following T_3_-induced metamorphosis. MMP9 is a metalloproteinase involved in the deconstruction of the extracellular matrix and is required for tail resorption (125). Following 100 nM TBBPA exposure in the context of T_3_-stimulated metamorphosis, *thra* mRNAs were significantly increased in the brain relative to TBBPA exposure alone while the abundance of *pcna* transcripts was decreased in the tail (75).

Fini et al. demonstrated that *X. laevis* tadpoles (NF stage 45) can rapidly take up radiolabeled TBBPA and retain the parent TBBPA and its biotransformed metabolites (TBBPA-glucuronide, TBBPA-sulfate, TBBA-glucuronide-sulfate), while only gradually releasing them (77). TBBPA was shown to be the principal agent of antithyroidal activity, not its biotransformed congeners. TBBPA, but not its metabolites, impaired T_3_-induced regression in tadpole head size and gills (77). Moreover, by using transgenic tadpoles containing a *thibz* promoter-green fluorescent protein (*thibz*-GFP) TH-response reporter construct, 10 μM TBBPA, not its sulfate conjugates, inhibited T_3_-induced GFP expression (77). TH metabolizing enzymes, deiodinases, UDP-glucuronyl transferases and sulfotransferases were not affected by TBBPA with or without T_3_ induction. A GAL4-luciferase reporter assay using the *Xenopus* TRα ligand binding domain transiently transfected into HeLa cells demonstrated that TBBPA can effectively compete with T_3_ binding. However, sulfated TBBPA conjugates lack this T_3_ displacement capacity (77). Independent studies in *X. laevis* recapitulated similar finding of TBBPA inhibition of T_3_-mediated reductions in head area, hindlimb length and decreased apoptosis and epithelial folds within intestines (78–80). Additionally, various groups described the restricted activation of a *thibz* response element-luciferase reporter assay and the reduction of tissue-specific gene expression of *thrb, st3, klf9, cebpd, mmp2, dio2*, and *dio3* transcripts upon TBBPA inhibition of T_3_-induced metamorphosis (Table 3) (78–80).

TBBPA is proposed to have developmental stage-specific effects on *X. laevis* metamorphosis, potentially related to endogenous levels of TH. During pre- and prometamorphosis, endogenous levels of TH are low and TBBPA exposure was associated with increased hindlimb length and the promotion of development. However, during metamorphic climax when TH amounts are maximal, developmental stage transitions were delayed (80). An additional potential confounder may be the amount of TBBPA that metamorphic anurans are exposed to (74). Molecular analysis of *Pelophylax (P.) nigromaculatus* intestines showed that tadpoles exposed to low concentrations of TBBPA (1 nM) had agonistic effects on T_3_-induced expression of TH-response genes (Table 3). In contrast, higher TBBPA concentrations (100–1,000 nM) had antagonistic effects in the same experimental paradigm (74). The molecular mechanisms by which TBBPA may act as both an agonist and antagonist of tissue-specific development while endogenous TH levels vary need to be ascertained.

### Bisphenol A (BPA)

Bisphenol A (4,4′ isopropylidenediphenol; BPA) is a widely used monomer in the manufacture of polycarbonate plastics, epoxy resins and food containers. More than 2.2 million metric tons of BPA were globally produced in 2009. Since the 1930's, BPA was known to be xenoestrogenic and growing concerns about the exposure of humans to BPA culminated in the US Food and Drug Administration banning BPA from baby bottles in 2012 (126). Despite debates between food and drug administrations and researchers about the endocrine disrupting effects of BPA, this monomer has been implicated in a plethora of etiologies including diabetes, obesity, and hypothalamic neuroendocrine dysfunction. Early developmental periods are also ostensibly sensitive to the effects of BPA (127–129).

BPA is found ubiquitously throughout the environment, soils, surface waters, sewage, and more. Detoxification of BPA within organisms occurs through glucuronidation and the biotransformed oxidative metabolites that result can have greater endocrine disrupting effects than the parent BPA or analog (130). While the effects of BPA on estrogenic dysregulation are well-studied, BPA can also affect signaling pathways of THs, androgens, and glucocorticoids (130). BPA exposure inhibits amphibian metamorphosis by targeting TH signaling and is extensively reviewed in Heimeier and Shi (131).

*X. laevis* embryos exposed to BPA displayed delayed metamorphosis by 2–4 stages at NF stages 52–54 (Table 3) (93). Tadpoles exposed to BPA had similarly delayed natural and T_4_-induced metamorphosis. Cultured tadpole tails treated with BPA had repressed T_3_-induced tail shortening and had BPA-inhibited *thrb* expression in the presence and absence of T_4_ stimulation (93).

Twenty-one day exposure of *X. laevis* tadpoles to BPA concentrations that were equivalent to human infant exposures also protracted T_3_-induced metamorphosis by 8 stages and stalled intestinal development (Table 3) (94). By 4 days, however, maladaptive molecular effects were observed in the reduced expression of early T_3_-responsive genes, *st3* and *thibz*, and the late responders, *mmp2* and *timp2*, in the intestine following combined BPA and T_3_ exposures. An oligo DNA microarray analysis of the intestinal transcriptome confirmed that BPA antagonizes the expression of genes involved in T_3_ signaling pathways (Table 3) (94).

### Genistein

Genistein is a plant-synthesized isoflavinoid found in high amounts in soy products (132). As a phytoestrogen, the endocrine disrupting capabilities of this compound have been well-studied for estrogen signaling [reviewed by Henley and Korach (133)]. However, its effects on TH signaling have been far less studied. Ji et al. acutely exposed premetamorphic *R. catesbeiana* tadpoles to T_3_ and then cultured the tail tips in the presence or absence of genistein to determine the effects of this contaminant on TH-induced metamorphic changes (82). Exposure to genistein led to the ablation of tail tip regression seen upon exposure to only T_3_. This morphological response is correlated with a decreased abundance of the *thrb* transcript (Table 3). In support of this finding, Hinther et al. also found decreased *thrb* upon exposure of cultured tail fin of *R. catesbeiana* to genistein, both induced and not induced by T_3_ (Table 3) (81). A possible mechanism by which TH signaling is being disturbed is through modulation of phosphorylation pathways. Genistein is a tyrosine protein kinase inhibitor (134), which is demonstrated in this amphibian model by leading to reduced overall tyrosine phosphorylation in T_3_-exposed *R. catesbeiana* tail tips cultured with genistein (82). As tyrosine phosphorylation of protein kinase C (PKC) is known to increase the activity of this kinase (135), the decreased tyrosine phosphorylation induced by genistein is correlated with negative PKC activity. It is postulated that this phosphorylation pathway impacts TH signaling through PKC serine phosphorylation of TRα. Upon acute exposure to T_3_, there is a significant increase in serine phosphorylation in *R. catesbeiana* tail tips, which can be reversed with PKC inhibitors (82). This response is attenuated by exposure to genistein, which likely leads to the observed decrease in the TH response gene *thrb*. Genistein can also affect thyroid peroxidase function in mammalian systems [reviewed by Doerge and Sheehan (136)]; however, whether this affects TH signaling in amphibians has yet to be determined. Further studies are needed to determine the role of phosphorylation pathways in cellular level TH signaling and whether other areas of the greater TH signaling pathway are affected by this contaminant.

### Phthalates

Phthalates are plasticizers added to increase the flexibility of plastics. These contaminants can be found in the air, soil, freshwater, and saltwater (137–139). The ubiquity of phthalates in the environment is concerning as they have shown to have TH disrupting effects [reviewed by Mathieu-Denoncourt et al. (140)]. Using a T_3_-activated *X. laevis* reporter cell system (Table 3), Sugiyama looked at the effects of five different phthalates on T_3_ signaling within the constructed cells (Table 3) and found di-n-butyl phthalate, n-butylbenzyl phthalate and dicyclohexyl phthalate caused a decrease in activity (95). These TH-disrupted responses were all associated with a decrease in endogenous *thrb* mRNAs in the reporter cells. N-butylbenzyl phthalate also led to decreased *thrb* with a T_3_-induced whole tadpole exposure. In line with these findings, Shen et al. found that chronic exposure of *X. laevis* tadpoles to di-n-butyl phthalate and its metabolite mono-n-butyl phthalate resulted in decreased *thrb* (96).

The mechanism by which phthalates disrupt TH signaling within the cell likely involves the regulation of TRs. Using a TR-mediated reporter gene assay, Shen et al. found that dibutyl phthalate, mono-n-butyl phthalate, and di-2-ethylhexyl phthalate demonstrated TRβ agonist activity (141). As TRs have various methods by which they can be regulated, Shen et al. queried the involvement of the TR corepressor silencing mediator for retinoid or TH receptors (SMRT) in the phthalate-dependent TR regulation and found that both di-n-butyl phthalate and mono-n-butyl phthalate increased the interaction between SMRT and TR in a mammalian two-hybrid assay (Table 3) (96). Furthermore, in the amphibian system, decreased methylation of the promoter region of *thrb* was found upon exposure to mono-n-butyl phthalate, which could be involved in TR-mediated regulation of the *thrb* gene. However, the same result was not seen with di-n-butyl phthalate, indicating potential differences in phthalate response (96). The involvement of other epigenetic mechanisms, such as histone post-translational modification, has yet to be elucidated. In contrast to the aforementioned studies, Mathieu-Denoncourt found that chronic exposure to monomethyl phthalate, a dimethyl phthalate metabolite, led to an increased metamorphic rate in *X. tropicalis* that associated with no TH response gene expression changes (Supplementary Table 1) (142). This suggests that various phthalates may have different mechanisms of disruption and/or the timing of TH response gene effects have differing response kinetics that were not captured in the study. Further work on these substances on a broader range of amphibian species is warranted.

### Metals

Metals acting as environmental contaminants stem from a variety of natural and anthropogenic sources (143). Heavy metals are notable environmental endocrine disrupting chemicals (EDCs) and can dysregulate TH-driven amphibian metamorphosis upon exposure.

Cadmium (Cd) exposure has been shown to significantly decrease metamorphosis in *B. americana* (144), as well as completely block completion of metamorphosis in other amphibians like *Pleurodeles waltl* (145). There is a significant correlation between Cd concentration and decreasing rates of metamorphosis in *X. laevis* (146). Furthermore, the effects of Cd exposure are exacerbated in male *X. laevis* tadpoles when the environmental pollutant estradiol-17β (E_2_) is present (147).

Sun et al. observed significant decreases in *dio2, thra*, and *thrb* transcripts following Cd exposures in *B. gargarizans* at concentrations an order of magnitude lower than previously reported to decrease metamorphic rate (83). At the lowest Cd concentration, an increase in *thra* expression was observed, but this may be due to using *actb* as a single normalizer, which can be TH-responsive (87). Thyroid histology revealed significant follicular cell hyperplasia in the cadmium-exposed animals.

Copper is naturally ubiquitous in the environment and influxes of anthropogenic copper occur due to soil disturbances or agricultural runoff (148). In several Ranidae species and *B. gargarizans*, chronic exposure to copper can significantly delay the rate of metamorphosis (Table 3) (85, 148, 149). Wang et al. showed that copper exposure in *B. gargarizans* significantly increased *dio3* expression and significantly decreased *dio2, thra*, and *thrb* expression at copper concentrations greater than what caused metamorphic delay (85). Although a transcriptional response is expected at lower concentrations, it is possible that measurements were done too late to observe significant changes in TH-related transcription as tadpole exposures commenced at Gosner stage 26 and transcript quantification did not occur until stages 42 and 46. Copper exposure also induced follicular cell hyperplasia in the thyroid gland.

Chronic mercury exposure exhibited a similar phenomenon in *B. gargarizans* as did copper; metamorphosis was delayed at lower concentrations than what caused significant decreases in *dio2, thra*, and *thrb* expression and induced follicular cell deformation in the thyroid gland (Table 3) (86). Again, transcript measurements were performed much later than the initial exposure such that lower concentration transcript effects may have been missed.

Other metals that resulted in a delay in metamorphosis include lead (Pb) in *R. pipiens* (Table 3), iron (Fe; ionized or ore particulates) or manganese (Mn) in *R. catesbeiana*, and depleted uranium (U) in *X. laevis* tadpoles (Table 3 and Supplementary Table 1) and further research on their effects on TH signaling is needed (150–152).

### Nanoparticles

Several metals have been manufactured as constituents of nanoparticles. Nanoparticles are any particles that have at least one dimension <100 nm (153). These nanoparticles possess unique properties compared to their ionic counterparts that make them highly desirable for wide use in industrial and medical applications. However, this has led to significant environmental contamination by nanoparticles and the endocrine disrupting potential of nanoparticles has been well-documented (153). As nanoparticles have unique aggregation and surface charge distributions, their exposure often results in different endocrine disrupting effects compared to their corresponding metal ions (154). It is important to study the endocrine disrupting potential of metal ions and nanoparticles separately as the effects of one are not necessarily predictive of the other. Nevertheless, few studies directly compare the effects of nanoparticle and metal ion exposures in the same study. Further complications in comparing the effects of nanoparticle and constituent ion exposures arise from differences in experimental conditions and species studied.

Chronic exposure to zinc, copper, and titanium oxide nanoparticles can delay metamorphosis in *X. laevis* tadpoles (155–158). However, titanium oxide-based nanoparticles or their ionic counterparts had no effect on TH signaling in the *R. catesbeiana* C-fin assay (159). Nanoparticle interference significantly decreased the rate of metamorphosis in *R*. *sylvatica* tadpoles chronically exposed to nanogold (Supplementary Table 1) (160).

Specific gene targets of nanoparticle endocrine disruption were investigated by Hinther et al. using a *R. catesbeiana* C-fin assay and 48 h exposures (84). They found that exposure to silver nanoparticles or Cd telluride quantum dots in combination with T_3_ significantly decreased the expression of the TH-responsive genes: *rlk1* and *thrb* (Table 3). The extent of TH-mediated gene disruption arising from 28 day nanosilver exposures was further evaluated by Carew et al. in pre- and prometamorphic *X. laevis* tadpoles (87). They found that, while exposure did not alter the overall rate of metamorphosis, there were transient perturbations of leg length and snout/vent length that were pre- or prometamorph-specific. Using a MAGEX cDNA array and qPCR performed on liver tissue extracted from these tadpoles, they identified 3 induced and 4 repressed transcripts in premetamorphs and 12 induced and 4 repressed transcripts in prometamorphs exposed to nanosilver (Table 3) (87). Of these, *mmp9, pparg*, and *trip4* have linkages to TH signaling pathways.

### Pesticides

#### Acetochlor

Acetochlor [2-chloro-*N*-(ethoxy-methyl)-*N*-(2-ethyl-6-methylphenyl) acetamide] is a widely used preemergent herbicide and persistent organic pollutant that contaminates groundwater (161). More than 10 million kg of acetochlor are used per year in the United States, with surface water concentrations ranging from median levels of 2.7 nM (730 ng/L) to as high as 10 nM (2.7 μg/L) within the 80th percentile of measurements sampled in the Midwestern United States (162, 163). Acetochlor can induce TH-dependent dysfunction and other pathologies in a variety of aquatic species (164–167). In combination with other pesticides, acetochlor may contribute to altered comorbid fungal infections in amphibians (168).

Concurrent treatment of premetamorphic *R. pipiens* tadpoles with acetochlor and T_3_ resulted in the acceleration of metamorphosis as evidenced by precocious forelimb emergence (169). As priming tadpoles with T_3_ prior to acetochlor treatment did not cause accelerated metamorphosis, it was concluded that acetochlor was interacting with T_3_ in a TR-independent manner to elicit precocious development (169).

*R. catesbeiana* tadpoles exposed to environmentally relevant concentrations of acetochlor (10 nM) did not affect *thrb* expression in tail fin biopsies (89). However, the combined treatment of acetochlor with T_3_ caused a synergistic increase in *thrb*, which concurred with earlier morphological findings of accelerated metamorphosis (89). Acetochlor induced the upregulation of *thra* and *thrb* in the brains of athyroid premetamorphic *R. catesbeiana* tadpoles and these increases were amplified upon exogenous T_3_ treatment (88). These results suggest a tissue-specific sensitivity to acetochlor. The *thra/thrb* transcript ratios were also altered and these transcript changes were not associated with any effects on escape behavior following acetochlor treatment (88).

Understanding of the TH-dependent molecular mechanisms disrupted by acetochlor was refined by cDNA microarray studies in *X. laevis*. Crump et al. demonstrated that changes in gene expression precede the morphological changes of T_3_-induced accelerated metamorphosis ensuing from acute and environmentally-relevant acetochlor exposures (90). After 48 h, acetochlor exposure caused a T_3_-mediated increase in *thra* and *thrb* and the overall magnification of genes otherwise upregulated by T_3_ (90). Of interest is that genes normally downregulated by T_3_ showed an attenuated response in the presence of acetochlor, suggesting that acetochlor perturbs mechanisms of transcriptional regulation (Table 3). Such impairment of transcription implies that acetochlor may disrupt epigenetic modes of regulation (90).

During prometamorphosis, endogenous levels of TH naturally increase and acetochlor exposure caused an accumulation of *thra* and *thrb* transcripts in tail fin biopsies from *R. catesbeiana* tadpoles. The brains of these acetochlor-treated prometamorphic tadpoles were assessed after a 59 day depuration period and no significant differences were observed in *thra* and *thrb* transcripts, although the ratios between them were altered at higher acetochlor concentrations (88). No major developmental changes were observed either in forelimb emergence, tail regression or mouth development (88).

#### Carbaryl

Carbaryl belongs to the carbamate class of insecticides and is commonly used in agricultural and home garden applications to control insect populations (170). Though presumed to have low toxicity, carbamates have structural similarities to organophosphate insecticides and can modify acetylcholinesterases, which has important implications for neurotransmission (171, 172). Carbaryl exposure can limit the resistance of amphibians to parasitic infection and its toxicity is exacerbated by previous Ranavirus infection of *R. sylvatica* (173, 174). Of outstanding interest are the implications for metamorphosing organisms in carbaryl-treated areas.

*R. clamitans* tadpoles exposed to environmentally relevant carbaryl concentrations did not have altered metamorphosis according to morphological metrics: tadpole development and time to metamorphosis (91, 175). However, both short- and long-term alterations in gene expression were observed in brain and tail tissues of tadpoles acutely exposed to carbaryl at 8 and 16 weeks post-hatching (Table 3) (91). Gosner stage 25 tadpoles exposed to carbaryl for 3 days at 16 weeks post-hatching had higher *thra* and *thrb* expression in the brain at Gosner stage 46. Greater *thrb* expression was also observed in tadpoles exposed at 8 weeks post-hatching (91). DNA microarray analysis highlighted the persistent transcript effects of carbaryl on altered brain pathways that included transcription, signal transduction and cell growth control. Immediately following carbaryl exposure, *thra* is increased in the tadpole tail (91). Pesticide exposures during such sensitive early developmental periods have potential consequences for fitness and health of the organism during its lifespan.

#### Glyphosate and Surfactants

Glyphosate is a commonly used herbicide for both domestic and agriculture applications around the world. Many commercially available formulations, such as Roundup^®^, contain glyphosate, which is rendered more toxic due to the inclusion of surfactants, whose toxicity can be influenced by pH, temperature, and species and developmental stage of exposed organisms (176, 177).

Several North American amphibians (*R. clamitans, R. pipiens, R. sylvatica*, and *B. americana*) exposed to glyphosate, different commercial herbicides and the surfactant polyethoxylated tallowamine (POEA) exhibited varying sensitivities depending on developmental stage and species (92). Glyphosate alone did not elicit deleterious effects, but in combination with POEA in Roundup Original^®^ and Roundup Transorb^®^, metamorphic defects were observed, particularly in *R. pipiens*, which was sensitive to these exposures (Table 3). Consequent to exposures at Gosner stage 25, tadpoles exhibited increased time to metamorphosis. Gonadal abnormalities were also observed as was tail damage that included necrosis, blistering, and abnormal growth (92). As observed with other disruptions to TH signaling, molecular aberrations were observed prior to phenotypic changes. At stage 25, but not 42, increases in *thrb* expression resulted from exposure to Roundup Original® and Roundup Transorb® (92). However, newer glyphosate herbicide formulations that do not include POEA are less toxic, making them more promising potential alternatives for agricultural and domestic use.

## Complex Mixtures

Although there is considerable focus on the effects of individual toxicants on TH activity, such chemicals do not persist alone in the environment. Mixture effects arising from the combination of different toxicants can result in TH-dependent disruptions not predicted by the individual chemical constituents (178).

### Metal Mixtures

Heavy metals exhibit increased toxicity as a consequence of mixture effects (179). Dorchin and Shanas examined the endocrine disrupting potential of a mixture of metals (Cu, Pb, and Ni) in concentrations comparable to that of runoff from busy highways (180). Exposure to this metal mixture significantly decreased the metamorphic rate of *Bufo* (*B*.) *viridis* tadpoles (Table 4) (180). A similar effect of metal mixtures was observed in *Limnodynastes peronei*, which exhibited a decreased rate in metamorphosis after being exposed to coal-mine wastewater containing low metal amounts (Table 4) (185).

**Table 4:** Summary of contaminant mixture effects on morphological and molecular endpoints for amphibians undergoing both natural and TH-induced metamorphosis.

		**Metamorphosis**		**Morphological/Behavioral**	**Molecular**			
***Chemical Mixture***	**Species**	**Natural**	**Induced**	**Results**	**Tissue**	**Technique**	**Result**	**References**
Bunker crude oil	*X. laevis*	Y			Whole tadpoles	qPCR	[0.25 g/L oil WAF] ↓*pparg, thrb*; [25 g/L oil WAF] ↑*pparg, dio2*	(181)
Refinery oil	*X. laevis*	Y			Whole tadpoles	qPCR	*↑pparg; ↓thrb*	(181)
Wastewater	*R. catesbeiana*		T_3_		C-fin^a^	qPCR	*↑thibz, thra, thrb*	(182, 183)
		Y		↓ predator cue avoidance	Olfactory epithelium	qPCR	*↑↓thibz*^b^*; ↑heket; ↓dio2*	(6, 36)
	*X. laevis*		T_3_	↑ metamorphic rate	Cultured tail	qPCR	*↑crhbp, dio2, fap, thrb*	(184)

a *Cultured tail fin assay*.

b *Varied response due to complex mixtures and treatment techniques*.

### Wastewater Effluents

Wastewater effluents (WWE) are complex mixtures that can contain contaminants from agricultural, industrial, and domestic sources and hence, can disrupt TH function. A primary source of contamination comes from PPCPs in human waste. Although wastewater goes through extensive filtration prior to dispersal, TH disruption still ensues from effluent exposures (36). The TH disruption potential of WWE was first examined in 2009 when Sowers et al. found that a 50% dilution of municipal WWE significantly decreased the rate of *R. pipiens* metamorphosis (Table 4) (186). A delay in metamorphosis was also observed in *R. catesbeiana* after exposure to pond water that had been a receptacle for municipal WWE (Table 4) (187).

Searcy et al. examined the effects on TH-mediated metamorphic gene expression within *X. laevis* tadpole *ex vivo* tail tip cultures exposed to WWE (184). Using oligo microarray and qPCR analyses, they found that WWE and T_3_ exposures significantly increased the expression of TH-sensitive genes: *thrb, dio2, crhbp*, and *fap* (Table 4). The *in vivo* effects of WWE on TH-linked gene expression was also demonstrated by Castillo et al. in a transgenic *X. laevis* harboring a *thibz*-GFP reporter construct that was activated by WWE exposure (Table 4) (188).

As wastewater treatments do not completely eliminate EDCs, Wojnarowicz et al. assessed the removal of EDCs by three methods of wastewater filtration using the C-fin assay (182). Despite clearing conventional contaminants, all three treatments produced WWEs that increased TH-sensitive gene expression (*thibz, thra, thrb*) upon exposure (Table 4). The treatment types also had conflicting results in their ability to clear TH signaling effects depending upon the season in which the WWEs were collected (Table 4) (182). In a later study, Wojnarowicz et al. demonstrated the inefficiency of municipal wastewater treatment plants by showing that there is little difference in the endocrine-disrupting potential of WWE to that of the original influent using TH-mediated molecular endpoints and C-fin assays (Table 4) (183).

The considerable compositional variation within WWEs poses a challenge when assessing their endocrine disrupting potential. Heerema et al. generated a wastewater standard composed of common PPCPs to evaluate the exposure effects of the simulated WWE and test the efficiency of wastewater treatment systems (36). After filtration using an anaerobic membrane bioreactor (AnMBR), the standard WWE induced a significant upregulation of TH-sensitive *thibz* in the olfactory epithelium of *R. catesbeiana* tadpoles. This suggests that the effluent was influencing TH-dependent pathways (Table 4). This study also assessed the behavioral effects, particularly predator cue avoidance, associated with WWE exposure. Once a tadpole is exposed to T_3_, it will stop responding to a simulated predator cue (36). WWE exposure mimicked the effects of T_3_ signaling in the olfactory epithelium and decreased predator cue avoidance (36). As a follow-up to this work, Jackman et al. showed that membrane enhanced biological phosphorous removal (MEBPR) performed better at removing EDCs from WWE than AnMBR (6). However, both effluent types resulted in the perturbation of TH-responsive gene transcript levels in the olfactory epithelium. TH agonist activity was observed in the AnMBR WWE and antagonist activity from MEBPR WWE, likely reflective of the influent source material (Table 4).

### Petroleum Oil Products

Oil spills from a variety of sources can contaminate freshwater systems, thereby affecting the local biota (189). Major toxic components of oils, such as napthenic acid (NA) and polycyclic aromatic hydrocarbons (PAHs), are dispersed within water-soluble fractions after a spill (190, 191). NA and PAHs act as EDCs in amphibians. NA can directly reduce the rate of metamorphosis in *X. tropicalis* and *R. pipiens* and PAHs from tar-based pavement sealers can significantly reduce the rate of metamorphosis in *X. laevis* (Supplementary Table 1) (192, 193). The endocrine disrupting potential of these compounds seems to be quite persistent in the environment as *R. sylvatica* tadpoles exposed to pond water from wetlands proximal to reclaimed oil sands had significantly altered T_3_/T_4_ ratios and had accelerated or delayed rates of metamorphosis depending on the age of the reclaimed wetland (Supplementary Table 1) (194).

The effects of NA and PAHs on TH-sensitive gene expression was evaluated by exposing *X. laevis* tadpoles to simulated oil spill conditions using water accommodated fractions (WAF). WAFs were prepared using bunker crude oil or refinery oil (181). Bunker crude WAF exposures resulted in a significant decrease in *dio2* and *thrb* and differential expression of *pparg* at various WAF concentrations (Table 4). Refinery WAF exposures resulted in a significant decrease in *thrb* expression and a significant increase in *pparg* expression (Table 4). Therefore, water soluble components of oil spills can adversely affect TH-sensitive gene expression critical for amphibian metamorphosis.

## Effects of Environmental Factors

### Temperature

Temperature serves as an important environmental cue for seasonality changes. As such, poikilothermic anurans have evolved to allow this environmental factor to serve as a critical cue in their developmental program. The role of temperature in modulating developmental timing is clearly demonstrated during natural metamorphosis when warmer temperatures lead to increased endogenous T_3_ and thereby a faster metamorphic rate (Supplementary Table 2) (195–198). Along with the increase in TH levels, there is an upregulation of TH-regulated transcripts (including *thra, thrb, thibz, dio2*, and *dio3*) that initiate the metamorphic program (Table 5) (195, 202).

**Table 5:** Effects of environmental factors on morphological and molecular endpoints for amphibians undergoing both natural and TH-induced metamorphosis.

			**Metamorphosis**		**Morphological**	**Molecular**			
***Category***	**Treatment**	**Species**	**Natural**	**Induced**	**Results**	**Tissue**	**Technique**	**Result**	**References**
Food restriction	Low food	*R. sylvatica*	Y		↓ metamorphic rate	Tail	qPCR	↓*thrb*	(80)
Pond drying	Decreasing water	*Pelobates cultripes*	Y		↑ metamorphic rate	Body without tail	qPCR	*↑thrb*	(199)
		*R. temporaria*	Y		↑ metamorphic rate	Liver	cDNA array (MAGEX 497 genes)	↑37↓1: cell cycle, transcription, integral to membrane	(200, 201)
			Y			Liver	qPCR	*↑cps1, thrb*	(200)
Temperature	High temperature	*R. catesbeiana*	Y			Liver	qPCR	*↑dio2, dio3, thibz, thrb*	(195)
				T_3_		Liver	qPCR	*↑thra, thrb*	(202)
	Low temperature	*R. catesbeiana*		T_3_	↓ tail regression	Back skin	qPCR	*↑klf9, thibz, thra; ↓dio2*	(65)
				T_3_		Brain	qPCR	*↓dio3, thra, thrb*	(65)
				T_3_		C-fin^a^	qPCR	*↓thra, thrb*	(65)
				T_3_		C-skin^b^	qPCR	*↓thra, thrb*	(65)
				T_3_		Liver	qPCR	*↓cps1, dio2, dio3, mmp11, nfic, otc, several energy metabolism transcripts, thibz, thra, thrb*	(65, 202–204)
				T_3_		Liver	ChIP	↓H3K36me3 in *thrb* gene	(204)
				T_3_		Liver	Thin layer chromatography	↓lipid polyunsaturation	(203)
				T_3_		Lung	qPCR	*↑klf9, thibz*	(65)
				T_3_		Plasma	Mutarotase-glucose oxidase method	Inhibit T_3_-induction of glucose	(203)
				T_3_		Tail fin	qPCR	*↑thibz*	(65)
UVBR	UVBR	*R. pipiens*	Y			Tail	qPCR	*↓dio2; ↑dio3*	(205, 206)

a *Cultured tail fin assay*.

b *Cultured back skin assay*.

Conversely, metamorphic rate slows as temperatures decrease and can be halted altogether at 4–5°C (Supplementary Table 1) (203, 207, 208). Although premetamorphic tadpoles will not undergo precocious TH-induced metamorphosis at cold temperatures, a TH-induced memory is established whereby the metamorphic program resumes when permissive temperatures are attained, even when no TH signal remains (207). This cold temperature arrest is observed at the transcriptomic level (Table 5) (65, 203, 204, 209). Hammond et al. induced metamorphosis at 5°C in premetamorphic *R. catesbeiana* tadpoles through T_3_ exposures and assessed transcript responses in the brain, liver, back skin, tail fin, and lung (65). Across all tissues, *thrb* did not show the rapid induction observed at permissive temperatures. Transcripts encoding the transcription factor *thibz*, however, were upregulated in response to T_3_ in all tissues, although this was not found in the liver by Suzuki et al. (Table 5) (203). Other TH response genes, including *dio2, dio3, cebp1, klf9*, and transcripts encoding urea cycle and energy metabolism enzymes showed varied responses across tissues, indicating there may be a tissue-specific response to T_3_ in cold temperatures (65, 202–204). Cold temperatures also inhibited the T_3_-induction of plasma glucose and decreased lipid polyunsaturation consistent with an effect on energy metabolism in tadpoles (Table 5) (203).

Regulation of chromatin structure is postulated to be a mechanism by which this differential gene expression occurs at permissive and non-permissive temperatures [reviewed by Hammond et al. (210)]. Using chromatin immunoprecipitation, Mochizuki et al. found that upon T_3_ exposure of *R. catesbeiana* at 4°C compared to 28°C, there was decreased association of positive transcription histone H3K36 marks within two known temperature responding genes in the liver: *thrb* and *cebp1* (Table 5) (204). How histone post-translational modifications may differentially regulate genes with upregulated transcription in cold temperatures and simultaneously establish a molecular memory of the TH signal to be activated under permissive conditions is unknown. As changing climate becomes an increasing threat to declining frog populations, it is critical to understand the effect that temperature has on the proper regulation of TH signaling during development.

### Ultraviolet B Radiation

Ultraviolet B radiation (UVBR) is becoming a growing concern as stratospheric ozone levels deplete (211). Paired with an increasing penetration of UVBR into the water column (212), embryo and tadpole stages, which reside in aquatic environments, are at a greater risk. Metamorphic or developmental consequences of UVBR exposure on these more sensitive stages varies depending on species and life stage exposures [reviewed in Croteau (205)]. Blocked or delayed postembryonic development is the most commonly seen defect upon UVBR exposure (Supplementary Table 1) (213, 214). Croteau et al. examined the relationship between UVBR-induced developmental delays in *R. pipiens* and whether it may be related to the disruption of TH signaling (206). Exposure to UVBR showed no effect on total body T_3_ or brain CRF levels, indicating that synthesis of TH through the HPT axis is unlikely affected, although total T_4_ was not measured (206). Rather, UVBR effects may act locally on peripheral tissues. Increased *dio3* found in the tail during stages preceding the observed morphological delay may cause decreased local TH through enhanced turnover (Table 5). There is also decreased expression of *dio2*, which may act to further regulate the activity of THs through decreased conversion of T_4_ to T_3_ (2). This local response highlights the need to look at various tissues as their response to UVBR may differ leading to uncoordinated metamorphosis. As levels of UVBR are expected to rise, it is imperative to determine its mechanism of action in TH disruption, especially for sensitive life stages like postembryonic development.

### Photocycle: Light-Dark Cycle Implications

Endocrine systems are entrained to the circadian clock. The TH axis is no exception, with THs following a rhythmic 24 h cycle (215). As photoperiod, along with temperature, is an environmental cue for seasonality changes, it is not surprising that many studies have found that the light:dark (L:D) cycle has an impact on metamorphic rate (Supplementary Table 1) (216–218). In the majority of studies, increasing photophase (light phase) of a 24 h L:D cycle or decreasing cycle length increases metamorphic response to TH stimulation (218). However, when the 24 h cycle is not maintained, the L:D ratio is no longer indicative of metamorphic rate. In a case where Wright et al. found decreased tail reduction with an increased photophase, there was 18L:12D, which surpasses the standard 24 h cycle (219).

The mechanism by which the L:D cycle alters TH-induced metamorphosis is poorly understood. It has been determined that altering the L:D cycle leads to differences in the fluctuating rhythm of T_4_ [reviewed by Wright (215)] (Supplementary Table 2). However, under any L:D cycle, there is an inextricable rise in T4 as development progresses until metamorphic climax (220). This indicates that the alteration in metamorphic rate is not due to a disruption in TH concentration. It is more likely that a disruption in the circadian rhythms of THs may lead to different interactions with agonists and antagonists (220, 221). It remains to be determined how this variation in cycling affects TH responses at the transcriptomic level, which may provide a better mechanistic understanding of how the L:D cycle impacts metamorphic timing.

### Pond Drying

Many tadpole species reside in ephemeral bodies of water. Loss of these temporary habitats is fatal to water-dwelling tadpoles; therefore, it is unsurprising that across species, there is a positive correlation between pond desiccation rate and speed of development into terrestrial frogs (Supplementary Table 1) (198, 222, 223). The ability to translate this environmental cue to a phenotypic response is proposed to occur through the HPT axis. CRF levels increase in response to habitat desiccation, preceding the morphological observation of hastened metamorphic rate (224). This stress-induced increase in CRF leads to augmented secretion of THs (Supplementary Table 2) (199, 224, 225), which can be reversed through exposure to CRF antagonists (225). This increase in activity in the HPT axis leads to accelerated metamorphosis through the downstream regulation of TH response genes. Johansson et al. used cDNA microarray and qPCR analyses to determine the hepatic transcriptomic response to simulated pond drying in *R. temporaria* (Table 5) (200). This study found that classic TH response genes, such as *thra* and *thrb*, increase along with decreasing water levels, which corroborates previous findings that decreased water levels lead to increased TH levels and higher expression of *thrb* in the blood (199). More liver-specific TH response transcripts also demonstrated significant changes, including urea cycle enzyme *cps1* (200). The ability of tadpoles to respond to decreasing water levels demonstrates the plasticity of TH-mediated metamorphosis that allows tadpoles to adapt to changing environments.

### Food Restriction

Similar to pond drying, availability of other resources, such as food, plays an important role in developmental timing. As metamorphosis often entails a niche transition from aquatic to terrestrial environments, it stands to reason that when resources available in the aquatic habitat are no longer sufficient, it may prompt a transition to a new environment where resources may be greater or competition lower. The impact of food restriction on metamorphosis has varied results (Supplementary Table 1) (226–231). Complete starvation and consistently low or actively decreasing food levels leads to increased metamorphic rate in *Scaphiopus* (*S*.) *hammondii* (226)*, Phrynobatrachus guineensis* (227), *and R. temporaria* (228). In contrast, consistently low or actively decreasing food sources reduces metamorphic rate in *Hyla cineria* and *Hyla gratiosa* (229, 230). The varied response may be due to the different life histories of the different species. Another contributing factor is the developmental timing of food restriction. D'Angelo et al. determined that there is a critical developmental point, around limb bud formation, before which metamorphosis will be stalled but after which, metamorphosis will be accelerated (232). Bulaeva et al. restricted food for *R. sylvatica* prior to this critical time point and found that the decrease in metamorphic rate coincided with a decrease in *thrb* transcript levels, indicating disrupted TH signaling (Table 5) (50). In contrast, histological analysis of the thyroid gland of the tadpoles starved past this critical period give evidence to a short burst of increased secretory activity (232). Evidence of this burst of thyroid activity was corroborated *in vitro* by Wright et al. who found a brief increase in secretion of T_4_ in cultured thyroids excised from *R. catesbeiana* tadpoles that were starved for 1 week compared to those that were fed consistently (Supplementary Table 2) (233). Boorse and Denver also found increased levels of T_3_ and T_4_ *in vivo* after food restriction in *S. hammondii* (234). Future studies have yet to determine how this burst of T_4_ affects the TH-induced transcriptomic program leading to increased metamorphic rate.

### Combined Chemical and Environmental Effects

It is well-established that individual environmental factors play an important role in the proper regulation of TH signaling during metamorphosis. In natural systems, however, temperature, photoperiod, UVBR intensity, and resource restriction (pond-drying and food restriction) effects are inextricably linked. Not only are they influenced by each other, but frogs are simultaneously exposed to all anthropogenic chemicals that enter their habitat. The combinatorial effect of environmental and chemical stressors can be exponentially more detrimental as they may work synergistically to increase toxicity or reduce an organism's capacity to respond to other stressors.

The additive toxicity of UVBR and various chemical contaminants has been well-documented [reviewed by Blaustein et al. (235) and Croteau et al. (205)]; however, the sublethal effects on development have not been as well-studied. Crump et al. found that environmentally-relevant levels of the estrogenic compound, octylphenol, had a combined effect with UVBR that increased metamorphic rate, unlike exposure to either factor individually (236). The TH-based mechanism by which this combined effect occurs was further studied by Croteau et al. who observed that at earlier developmental stages, there is a significant increase in *thrb* upon exposure to UVBR combined with octylphenol compared to either factor alone (Table 6) (206). This indicated that TH-signaling during metamorphosis is being differentially affected by the combination of chemical and environmental factors.

**Table 6:** Combined effects of chemical contaminants and environmental factors on morphological and molecular endpoints for amphibians undergoing both natural and TH-induced metamorphosis.

	**Treatment**			**Metamorphosis**		**Morphological**	**Molecular**			
***Category***	**Environmental**	**Chemical**	**Species**	**Natural**	**Induced**	**Results**	**Tissue**	**Technique**	**Result**	**References**
Temperature	28°C	Diuron	*R. catesbeiana*	Y			Liver	qPCR	*↑thibz*	(195)
	34°C		*R. catesbeiana*	Y			Liver	qPCR	*↑dio2, klf9*	(195)
	5°C	Ibuprofen	*R. catesbeiana*		T_3_		C-skin	qPCR	*↑klf9*	(65)
	5°C	Triclosan	*R. catesbeiana*		T_3_		C-skin	qPCR	*↑klf9*	(65)
	5°C to 24°C		*R. catesbeiana*		T_3_		C-fin	qPCR	*↑thrb*	(65)
UVBR	UVBR	4-Tert-octylphenol	*R. pipiens*	Y		↓ metamorphic rate	Tail	qPCR	*↑thrb*	(236)

Increases in metamorphic rate induced by warmer temperatures are compounded by concomitant contaminant exposures that also induce metamorphosis. Freitas et al. exposed *R. catesbeiana* tadpoles to the pesticide diuron and its metabolite, 3,4-dichloroaniline, at 28 and 34°C and observed an increased developmental response to both chemicals at the higher temperature compared to either exposure at the lower temperature or the temperature-matched control (Table 6) (195). This combination of exposures increased the expression of *dio2* and the warm temperature plus diuron exposure increased *klf9*, which likely explains the observed change in metamorphic rate.

Contaminants can also have an impact on the ability of environmental cues to regulate developmental timing. For species that overwinter as tadpoles, transitioning while the temperatures are still too low could be fatal. Hammond et al. investigated the impact of two known TH EDCs discussed above, ibuprofen, and TCS, on the temperature-controlled TH response in premetamorphic *R. catesbeiana* tadpoles (Table 6) (28, 29, 65). After a 48 h exposure to each contaminant at 5°C, both produced a significant increase in *klf9* in a cultured *R. catesbeiana* back skin biopsy assay (C-skin). In contrast, when tail fin biopsies from the same tadpoles were cultured in a C-fin assay, exposed to the chemicals at 5°C and then shifted into clean media at more permissive temperatures, there was a significant decrease in *thrb* after TCS exposure (Table 6). This indicates that both EDCs have the potential to disrupt proper TH signaling during the cold-induced establishment of TH molecular memory. As well, TCS exposure at cold temperatures may be remembered when warmer temperatures occur, potentially leading to detrimental effects throughout metamorphosis.

Natural systems contain combinations of environmental factors and chemical contaminants. It is therefore important to conduct studies with multiple stressors to provide more meaningful information. A changing climate and intensifying UVBR combined with increased anthropogenic contamination are escalating the need to elucidate how these factors influence critical biological systems such as TH signaling in metamorphosis, both independently and in combination with each other.

## Concluding Considerations

As our understanding of the disruption of TH-dependent metamorphosis by environmental and chemical perturbations improves, it is apparent that there are several pressing challenges that must be addressed. Anurans are keystone sentinel species that can portend the deleterious and combinatorial effects of contaminants and changing climate effects on all trophic levels within different environmental niches. While it is important to understand the mechanisms affected by a single contaminant, the environmental context in which the exposure occurs must be considered. Within an affected environment, disruption of TH-dependent metamorphosis is rarely, if ever, derived from an isolated contaminant. To this end, the interplay between environmental conditions and complex mixtures should be assessed in tandem to ascertain the cumulative effects on TH-dependent metamorphosis.

Amphibian screening assays have been developed that address the need for the timely detection of contaminants that affect TH-dependent metamorphosis and that accurately reflect *in vivo* changes (237). The *Xenopus* metamorphosis assay (XEMA) was initially conceived to assay the TH-disrupting capacity of compounds and derivatives of XEMA have since been developed (40, 78, 238). Similarly, cell lines and serum-free organ culture techniques, including tail fin (C-fin) and back skin (C-skin), have utility in ascertaining TH disrupting effects (81, 209, 239, 240). Organ culture techniques are particularly useful since they retain the three-dimensional structure of tissues while facilitating a repeated-measures analysis, including the rapid assessment of TH-dependent molecular changes in gene expression. As such, organ cultures provide an informative and complementary counterpart to conventional morphological and histological assessments (89). As changes in gene expression typically precede morphological variations during metamorphosis, transcriptomic assessments provide a more timely and sensitive assessment of altered metamorphic trajectories that may not be readily observed as a pathological phenotype. Non-lethal molecular assessments of tail fin biopsies are additionally well-suited to long-term studies involving repeated measures (84).

Careful consideration should be paid to the selection of amphibian species used to assess the ramifications of environmental contaminants on metamorphic dysfunction. Although *Xenopus* species are widely accepted animal models in research laboratories, their natural habitats, life cycles and physiologies are quite distinct from other anurans, such as Ranids, Hylids, and Bufonids (241). Consequently, physiological responses to environmental or chemical perturbations can differ widely between anurans. Therefore, the adoption of amphibian models that closely resemble native species in affected areas would provide the most meaningful assessments of TH-dependent metamorphic disruptions (242).

The use of qPCR, DNA microarrays, and RNA-seq in conjunction with morphological characterizations have demonstrated the sensitive and differential tissue-specific gene expression arising from environmental or toxicant exposures during TH-dependent metamorphosis (23, 32, 34, 209, 243). As cutting-edge ‘omics techniques—transcriptomics, genomics, epigenomics, proteomics, and metabolomics—are increasingly utilized with bioinformatics in ecotoxicology studies, it will be possible to elucidate the mechanisms affected by TH-disrupted metamorphosis in a comprehensive manner (210, 244–247). Considerable progress has been made in recent years with the sequencing of the *X. laevis, X. tropicalis, Nanorana parkeri*, and *R. catesbeiana* genomes and increasing numbers of transcriptomes; all of which are invaluable resources (247–251). Thoughtful consideration of the species, tissue-specificity, and developmental stage observed; the timing and duration of exposure and study conditions will be indispensable in establishing large-scale studies for meaningful meta-analyses.

Additional attention should be paid to the examination of metabolized derivatives that may be more potent than the parent congener. Biotransformed derivatives can be generated through metabolic activities within the affected organism or by physical transformation in the environment (for instance, weathering or photo-oxidation) (252–254). Compared to the parent congener, activated derivatives may consequently be more stable, better able to mimic or target different aspects of TH regulation (i.e., TH receptors, metabolizing enzymes, etc.) or be rendered more lipophilic, which would facilitate their uptake or excretion. The mechanisms underlying increased derivative toxicity, whether they are independent of or compromise TH regulation, is an important area of study.

With more than 70% of ~7,000 extant amphibian species threatened and declining around the world, there is an urgent need to address how anthropogenically-derived environmental disruptions are affecting vulnerable species (255). Humans are not impervious to the deleterious changes affecting wildlife; metabolomic studies demonstrated that metabolites altered during anuran metamorphosis are also associated with human disease outcomes (245). Moreover, a recent study demonstrated that TH-related gene expression and early brain development were altered in *X. laevis* following exposure to concentrations of chemicals (including TCS, phthalates, pesticides, and others) detected in human amniotic fluid (256). Given the developmental parallels between TH-dependent amphibian metamorphosis and mammalian postembryonic development, it is apparent that exposures negatively affecting amphibians will also impair human health (257). As sobering as these ramifications are, such deleterious outcomes are not necessarily irrevocable if timely remediation actions are taken. The genomic plasticity afforded by epigenetic alterations, while able to endure maladaptive stresses, similarly has the posited capacity to adapt to remediation. Such potentially ameliorative effects merit further investigation that would be best addressed using the genomics-based approaches discussed in conjunction with morphological analyses. Remediation efforts will require understanding the complexity of the ecological stresses and the interplay between complex toxicant mixtures and changing environmental conditions. The unique sensitivity of anurans to TH ideally positions them as indicators for not only metamorphic and developmental effects, but also for the fitness and reproductive success of all vertebrates that depend upon TH function.

## Author Contributions

All authors contributed to the conceptualization, literature research and interpretation, writing, and editing of the manuscript.

### Conflict of Interest Statement

The authors declare that the research was conducted in the absence of any commercial or financial relationships that could be construed as a potential conflict of interest.
