# Corrigendum: Contaminant and Environmental Influences on Thyroid Hormone Action in Amphibian Metamorphosis

**DOI:** 10.3389/fendo.2019.00405

**Published:** 2019-06-26

**Authors:** Anita A. Thambirajah, Emily M. Koide, Jacob J. Imbery, Caren C. Helbing

**Affiliations:** Department of Biochemistry and Microbiology, University of Victoria, Victoria, BC, Canada

**Keywords:** thyroid hormone, environmental contaminant, endocrine disruptor, frog tadpole, metamorphosis, environmental factors, transcriptomics, genomics

In the original article, there was a mistake in Figure 1 and the corresponding figure legend as published. The progression of tail regression and mouth sculpting relative to metamorphic timing was incorrectly depicted and there was an error in the reference in the figure legend. The corrected Figure 1 appears below.

**Figure 1:**
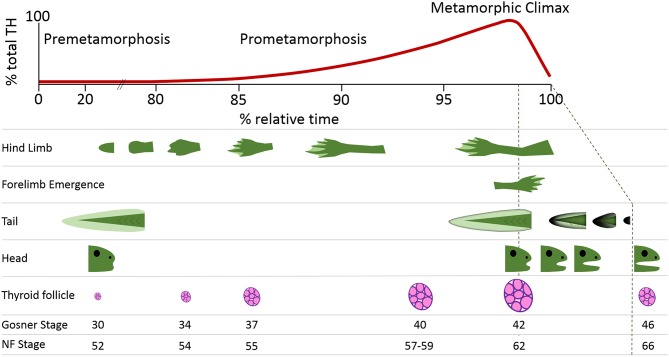
Thyroid hormone (TH) levels and key morphological hallmarks during frog postembryonic development. Amphibian metamorphosis is a postembryonic process driven by TH signaling. The free-swimming tadpole (0% relative time) has virtually undetectable levels of TH. The morphological changes that occur in the development of a tadpole to a juvenile frog (100% relative time) are inextricably aligned to internal rises in TH levels. These rising TH levels lead to progression through the stages of development, which can be seen through morphometric measurements including hindlimb development, forelimb emergence, tail regression, head shape changes, and thyroid follicle production. The Gosner and Nieuwkoop and Faber (NF) staging system comparisons are from Just (3).

The corrected **Figure 1** legend is “Thyroid hormone (TH) levels and key morphological hallmarks during frog postembryonic development. Amphibian metamorphosis is a postembryonic process driven by TH signaling. The free-swimming tadpole (0% relative time) has virtually undetectable levels of TH. The morphological changes that occur in the development of a tadpole to a juvenile frog (100% relative time) are inextricably aligned to internal rises in TH levels. These rising TH levels lead to progression through the stages of development, which can be seen through morphometric measurements including hindlimb development, forelimb emergence, tail regression, head shape changes, and thyroid follicle production. The Gosner and Nieuwkoop and Faber (NF) staging system comparisons are from Just (3).”

In the original article, reference 3 was incorrectly written as Gosner KL. A Simplified Table for staging anuran embryos and larvae with notes on identification. *Herpetologica*. (1960) 16:183–90. It should be Just, JJ, Kraus-Just J, Check DA. Survey of chordate metamorphosis. In: Gilbert LI, Frieden E, editors. *Metamorphosis*. Boston, MA: Springer (1981). p. 265–326.

The authors apologize for these errors and state that this does not change the scientific conclusions of the article in any way. The original article has been updated.

