# Supplementary material for: Contaminant and Environmental Influences on Thyroid Hormone Action in Amphibian Metamorphosis
Source: Front Endocrinol (Lausanne). 2019 May 14;10:276. doi: 10.3389/fendo.2019.00276 (PMC6530347; doi:10.3389/fendo.2019.00276)
Supplement: Supplementary file 1 [file Data_Sheet_1.PDF]

Supplementary Information

Contaminant and environmental influences on thyroid hormone action in amphibian metamorphosis

Anita A. Thambirajah[1,2], Emily M. Koide[1,2], Jacob J. Imbery[2] and Caren C. Helbing[2,*]

[1]These authors contributed equally to this work.

[2]Department of Biochemistry and Microbiology, University of Victoria, 3800 Finnerty Road, Victoria, British Columbia, V8P 5C2, Canada

*Correspondence:
Caren C. Helbing
E-mail: chelbing@uvic.ca

Supplementary Table 1. Effects of chemical contaminants and environmental factors on both natural and TH-induced metamorphic rate of amphibians

Category	Chemical	Species	Metamorphosis Natural	Metamorphosis Induced	Metamorphic rate	References
Industrial-Agricultural						
Flame retardants	Apolar sediment extract from polluted sites	*X. laevis*	Y		↓	(1,2)
	BDE-47	*X. tropicalis*	Y		↓	(3)
	BDE-99	*X. tropicalis*	Y		↓	(3)
	Clophen A50 (technical PCB mixture)	*R. temporaria*	Y		↓	(4)
	Clophen A50 (technical PCB mixture)	*X. laevis*	Y		↓	(1,2)
	DE-71	*R. pipiens*	Y		↓	(5)
		X. laevis	Y		↓	(6)
	PCB 126	*X. laevis*	Y		↓	(4)
	PCB 77	*X. laevis*	Y		↓	(1)
	PentaBDE DE-71	*X. laevis*	Y		↓	(6)
	TBBPA	*R. rugosa*		T_3	↓	(7)
		X. laevis	Y		↓	(8)
Metals	Al	*R. sylvatica*	Y		↓	(9)
	Cd	*B. americana*	Y		↓	(10)
		Pleurodeles waltl	Y		X	(11)
		X. laevis	Y		↓	(12)
	Cu	*R. pipiens*	Y		↓	(13)
		R. sylvatica	Y		↓	(9)
	Fe	*R. catesbeiana*	Y		↓	(14)
	Mn	*R. catesbeiana*	Y		↓	(14)
	Nanocopper oxide	*X. laevis*	Y		↓	(15)
	Nanogold	*R. sylvatica*	Y		↓	(16)
	Nanozinc oxide	*R. sylvatica*	Y		↓	(17)
	Pb	*R. pipiens*	Y		↓	(18)
	U (depleted)	*X. laevis*	Y		↓	(19)
Perchlorate	Ammonium perchlorate	*X. laevis*	Y		↓	(20,21)
	Sodium perchlorate	*R. sylvatica*	Y		↓	(22)
		X. laevis	Y		↓	(23–25)
Pesticide	Acetechlor	*R. pipiens*		T_3	↑	(26)
Plastics Additive	BPA	*X. laevis*	Y		↓	(27)
				T_4	↓	(27)
	Monomethyl phthalate	*X. tropicalis*		T_3	↑	(28)
PPCPs						
Estrogen	E_1	*B. bufo*	Y		↑	(29)
	E_2	*X. laevis*		T_3	↓	(12,30)
	EE_2	*R. pipiens*	Y		↓	(31)

		R. temporaria	Y		↓	(32)
		X. laevis	Y		↓	(33)
Mixtures						
Heavy metals	Synthetic road runoff (metal mixture)	*B. viridis*	Y		↓	(34)
PAH	Oil sands tailings-affected wetlands	*R. sylvatica*	Y		[Wetland reclamation <7 years] ↓; [Wetland reclamation >7 years] ↑	(35)
	Pavement sealer	*X. laevis*	Y		↓	(36)
Environmental						
Food restriction	Decreasing food	*S. couchii*	Y		↑	(37)
	Low food	*H. cineria*	Y		↓	(38,39)
		H. gratiosa	Y		↓	(38,39)
		Phrynobatrachus guineensis	Y		↑	(40)
		R. temporaria	Y		↑	(41)
		S. couchii	Y		↓	(37)
	Starvation	*S. hammondii*	Y		↑	(42)
Photoperiod	↑ Light:dark ratio in 24h cycle	*R. pipiens*	Y		↑	(43)
				T_4	↑	(44)
		X. laevis	Y		↓	(45)
	↓ Cycle length with equal light:dark ratio	*R. pipiens*	Y		↑	(44)
				T_4	↑	(44)
Pond drying	Decreasing water level	*H. pseudopuma*	Y		↑	(46)
		R. aurora	Y		↑	(47)
		S. hammondii	Y		↑	(42)
		S. intermontana	Y		↑	(47)
Temperature	High temperature	*B. boreas*	Y		↑	(48)
		Pseudacris regilla	Y		↑	(47)
		R. aurora	Y		↑	(47)
		R. pipiens	Y		↑	(49)
		S. intermontana	Y		↑	(47)
	Low temperature	*R. grylio*		T_3	X	(50)
		R. pipiens		T_4	X	(51)
UVBR	UVBR	*R. clamitans*	Y		X	(52)
		R. temporaria	Y		↓	(53)

Supplementary Table 2. Effects of chemical contaminants and environmental factors on TH levels in natural amphibian metamorphosis

Category	Chemical	Species	Tissue	Technique	Result	References
Industrial-Agricultural						
Perchlorate	Ammonium perchlorate	*X. laevis*	Whole tadpole	Radioimmunoassay	↓T_4	(20)
	Sodium perchlorate	*X. laevis*	Cultured thyroid and pituitary	ELISA	↓T_4	(54)
			Blood	HPLC/ICP-MS	↓T_4	(55)
			Thyroid	HPLC/ICP-MS	↓T_4	(55)
PPCPs						
Estrogen	Diethylstilbestrol	*R. catesbeiana*	Purified recombinant TTRs	Competitive binding assay	Major↓T_3 binding	(56)
		X. laevis	Purified recombinant TTRs	Competitive binding assay	Major↓T_3 binding	(56)
	E_2	*R. catesbeiana*	Plasma	Competitive binding assay	Minor↓T_3 binding	(56)
		R. ridibunda	Plasma and kidney	Radioimmunoassay	↓T_3	(57)
			Plasma	Radioimmunoassay	↓T_4	(57)
			Plasma	Radioimmunoassay	↓T_3:T_4 ratio	(57)
	EE_2	*R. temporaria*	Whole tadpole	ELISA	No change inT3	(32)
Mixtures						
PAH	Oil sands tailings-affected wetlands	*R. sylvatica*	Whole tadpole	ELISA	↓T_3:T_4 ratio	(35)
Environmental						
Food restriction	Starvation	*R. catesbeiana*	Thyroid gland	Radioimmunoassay	↑T_4 secretion	(58)
		S. hammondii	Whole tadpole	Radioimmunoassay	↑T_3	(59)
			Whole tadpole	Radioimmunoassay	↑T_4	(59)
Photoperiod	Δ Light:dark ratio in 24h cycle	*R. catesbeiana*	Plasma	Radioimmunoassay	Δ T_4 cycling	
Pond drying	Decreasing water level	*S. hammondii*	Whole tadpole	Radioimmunoassay	↑T_3	(59)
			Whole tadpole	Radioimmunoassay	↑T_3	(42)
			Whole tadpole	Radioimmunoassay	↑T_4	(42)
		Pelobates cultripes	Tail	Radioimmunoassay	↑T_4	(60)
Temperature	High temperature	*R. catesbeiana*	Plasma	ELISA	↑T_3	(61)
Environmental + Chemical						
Temperature	28°C + polybrominated diphenyl ethers	*R. pipiens*	Whole tadpole	EIA	↓T_3	(49)
	34°C + 3,4-dichloroaniline	*R. catesbeiana*	Blood	ELISA	↓T_3	(61)
	34°C + Diuron		Blood	ELISA	↑T_3	(61)

References

1. Gutleb AC, Mossink L, Schriks M, van den Berg HJH, Murk AJ. Delayed effects of environmentally relevant concentrations of 3,3′,4,4′-tetrachlorobiphenyl (PCB-77) and non-polar sediment extracts detected in the prolonged-FETAX. *Sci Total Environ* (2007) **381**:307–315. doi:10.1016/j.scitotenv.2007.03.002

2. Gutleb AC, Schriks M, Mossink L, Berg JHJ van den, Murk AJ. A synchronized amphibian metamorphosis assay as an improved tool to detect thyroid hormone disturbance by endocrine disruptors and apolar sediment extracts. *Chemosphere* (2007) **70**:93–100. doi:10.1016/j.chemosphere.2007.06.048

3. Carlsson G, Kulkarni P, Larsson P, Norrgren L. Distribution of BDE-99 and effects on metamorphosis of BDE-99 and -47 after oral exposure in *Xenopus tropicalis*. *Aquat Toxicol* (2007) **84**:71–79. doi:10.1016/j.aquatox.2007.06.003

4. Gutleb AC, Appelman J, Bronkhorst M, van den Berg JHJ, Murk AJ. Effects of oral exposure to polychlorinated biphenyls (PCBs) on the development and metamorphosis of two amphibian species (*Xenopus laevis* and *Rana temporaria*). *Sci Total Environ* (2000) **262**:147–157. doi:10.1016/S0048-9697(00)00598-2

5. Coyle TLC, Karasov WH. Chronic, dietary polybrominated diphenyl ether exposure affects survival, growth, and development of *Rana pipiens* tadpoles. *Environ Toxicol Chem* (2010) **29**:133–141. doi:10.1002/etc.21

6. Balch GC, Vélez-Espino LA, Sweet C, Alaee M, Metcalfe CD. Inhibition of metamorphosis in tadpoles of *Xenopus laevis* exposed to polybrominated diphenyl ethers (PBDEs). *Chemosphere* (2006) **64**:328–338. doi:10.1016/j.chemosphere.2005.12.019

7. Kitamura S, Kato T, Iida M, Jinno N, Suzuki T, Ohta S, Fujimoto N, Hanada H, Kashiwagi K, Kashiwagi A. Anti-thyroid hormonal activity of tetrabromobisphenol A, a flame retardant, and related compounds: Affinity to the mammalian thyroid hormone receptor, and effect on tadpole metamorphosis. *Life Sci* (2005) **76**:1589–1601. doi:10.1016/j.lfs.2004.08.030

8. Zhang Y-F, Xu W, Lou Q-Q, Li Y-Y, Zhao Y-X, Wei W-J, Qin Z-F, Wang H-L, Li J-Z. Tetrabromobisphenol A disrupts vertebrate development via thyroid hormone signaling pathway in a developmental stage-dependent manner. *Environ Sci Technol* (2014) **48**:8227–8234. doi:10.1021/es502366g

9. Peles JD. Effects of chronic aluminum and copper exposure on growth and development of wood frog (*Rana sylvatica*) larvae. *Aquat Toxicol Amst Neth* (2013) **140**–**141**:242–248. doi:10.1016/j.aquatox.2013.06.009

10. James SM, Little EE. The effects of chronic cadmium exposure on American toad (*Bufo americanus*) tadpoles. *Environ Toxicol Chem* (2003) **22**:377–380.

11. Flament S, Kuntz S, Chesnel A, Grillier-Vuissoz I, Tankozic C, Penrad-Mobayed M, Auque G, Shirali P, Schroeder H, Chardard D. Effect of cadmium on gonadogenesis and metamorphosis in *Pleurodeles waltl* (urodele amphibian). *Aquat Toxicol Amst Neth* (2003) **64**:143–153.

12. Sharma B, Patiño R. Effects of cadmium, estradiol-17beta and their interaction on gonadal condition and metamorphosis of male and female African clawed frog, *Xenopus laevis*. *Chemosphere* (2010) **79**:499–505. doi:10.1016/j.chemosphere.2010.02.044

13. Chen T-H, Gross JA, Karasov WH. Adverse effects of chronic copper exposure in larval northern leopard frogs (*Rana pipiens*). *Environ Toxicol Chem* (2007) **26**:1470–1475.

14. Veronez AC da S, Salla RV, Baroni VD, Barcarolli IF, Bianchini A, dos Reis Martinez CB, Chippari-Gomes AR. Genetic and biochemical effects induced by iron ore, Fe and Mn exposure in tadpoles of the bullfrog *Lithobates catesbeianus*. *Aquat Toxicol* (2016) **174**:101–108. doi:10.1016/j.aquatox.2016.02.011

15. Nations S, Long M, Wages M, Maul JD, Theodorakis CW, Cobb GP. Subchronic and chronic developmental effects of copper oxide (CuO) nanoparticles on *Xenopus laevis*. *Chemosphere* (2015) **135**:166–174. doi:10.1016/j.chemosphere.2015.03.078

16. Fong PP, Thompson LB, Carfagno GLF, Sitton AJ. Long-term exposure to gold nanoparticles accelerates larval metamorphosis without affecting mass in wood frogs (*Lithobates sylvaticus*) at environmentally relevant concentrations. *Environ Toxicol Chem* (2016) **35**:2304–2310. doi:10.1002/etc.3396

17. Nations S, Long M, Wages M, Canas J, Maul JD, Theodorakis C, Cobb GP. Effects of ZnO nanomaterials on *Xenopus laevis* growth and development. *Ecotoxicol Environ Saf* (2011) **74**:203–210. doi:10.1016/j.ecoenv.2010.07.018

18. Chen T-H, Gross JA, Karasov WH. Sublethal effects of lead on northern leopard frog (*Rana pipiens*) tadpoles. *Environ Toxicol Chem* (2006) **25**:1383–1389. doi:10.1897/05-356R.1

19. Mitchell SE, Caldwell CA, Gonzales G, Gould WR, Arimoto R. Effects of depleted uranium on survival, growth, and metamorphosis in the African clawed frog (Xenopus laevis). *J Toxicol Environ Health A* (2005) **68**:951–965. doi:10.1080/15287390590912595

20. Goleman WL, Carr JA, Anderson TA. Environmentally relevant concentrations of ammonium perchlorate inhibit thyroid function and alter sex ratios in developing *Xenopus laevis*. *Environ Toxicol Chem* (2002) **21**:590–597. doi:10.1002/etc.5620210318

21. Goleman WL, Urquidi LJ, Anderson TA, Smith EE, Kendall RJ, Carr JA. Environmentally relevant concentrations of ammonium perchlorate inhibit development and metamorphosis in *Xenopus laevis*. *Environ Toxicol Chem* (2002) **21**:424–430. doi:10.1002/etc.5620210227

22. Bulaeva E, Lanctôt C, Reynolds L, Trudeau VL, Navarro-Martín L. Sodium perchlorate disrupts development and affects metamorphosis- and growth-related gene expression in tadpoles of the wood frog (*Lithobates sylvaticus*). *Gen Comp Endocrinol* (2015) **222**:33–43. doi:10.1016/j.ygcen.2015.01.012

23. Opitz R, Schmidt F, Braunbeck T, Wuertz S, Kloas W. Perchlorate and ethylenethiourea induce different histological and molecular alterations in a non-mammalian vertebrate model of thyroid goitrogenesis. *Mol Cell Endocrinol* (2009) **298**:101–114. doi:10.1016/j.mce.2008.08.020

24. Zhang F, Degitz SJ, Holcombe GW, Kosian PA, Tietge J, Veldhoen N, Helbing CC. Evaluation of gene expression endpoints in the context of a *Xenopus laevis* metamorphosis-based bioassay to detect thyroid hormone disruptors. *Aquat Toxicol* (2006) **76**:24–36. doi:10.1016/j.aquatox.2005.09.003

25. Ruthsatz K, Dausmann KH, Drees C, Becker LI, Hartmann L, Reese J, Sabatino NM, Peck MA, Glos J. Altered thyroid hormone levels affect body condition at metamorphosis in larvae of *Xenopus laevis*. *J Appl Toxicol* (2018) **38**:1416–1425. doi:10.1002/jat.3663

26. Cheek AO, Ide CF, Bollinger JE, Rider CV, McLachlan JA. Alteration of leopard frog (*Rana pipiens*) metamorphosis by the herbicide acetochlor. *Arch Environ Contam Toxicol* (1999) **37**:70–77.

27. Iwamuro S, Sakakibara M, Terao M, Ozawa A, Kurobe C, Shigeura T, Kato M, Kikuyama S. Teratogenic and anti-metamorphic effects of bisphenol A on embryonic and larval *Xenopus laevis*. *Gen Comp Endocrinol* (2003) **133**:189–198. doi:10.1016/S0016-6480(03)00188-6

28. Mathieu-Denoncourt J, de Solla SR, Langlois VS. Chronic exposures to monomethyl phthalate in Western clawed frogs. *Gen Comp Endocrinol* (2015) **219**:53–63. doi:10.1016/j.ygcen.2015.01.019

29. Frieden E, Naile B. Biochemistry of Amphibian Metamorphosis: 1. Enhancement of induced metamorphosis by glucocorticoids. *Science* (1955) **121**:37-39.

30. Gray KM, Janssens PA. Gonadal hormones inhibit the induction of metamorphosis by thyroid hormones in *Xenopus laevis* tadpoles in vivo, but not in vitro. *Gen Comp Endocrinol* (1990) **77**:202–211. doi:10.1016/0016-6480(90)90304-5

31. Hogan NS, Duarte P, Wade MG, Lean DRS, Trudeau VL. Estrogenic exposure affects metamorphosis and alters sex ratios in the northern leopard frog (*Rana pipiens*): Identifying critically vulnerable periods of development. *Gen Comp Endocrinol* (2008) **156**:515–523. doi:10.1016/j.ygcen.2008.03.011

32. Brande-Lavridsen N, Christensen-Dalsgaard J, Korsgaard B. Effects of ethinylestradiol and the fungicide prochloraz on metamorphosis and thyroid gland morphology in *Rana temporaria*. *Open Zool J* (2010) **3**:7–16. doi:10.2174/1874336601003020007

33. Tompsett AR, Wiseman S, Higley E, Pryce S, Chang H, Giesy JP, Hecker M. Effects of 17α-ethynylestradiol on sexual differentiation and development of the African clawed frog (*Xenopus laevis*). *Comp Biochem Physiol Part C Toxicol Pharmacol* (2012) **156**:202–210. doi:10.1016/j.cbpc.2012.06.002

34. Dorchin A, Shanas U. Assessment of pollution in road runoff using a *Bufo viridis* biological assay. *Environ Pollut* (2010) **158**:3626–3633. doi:10.1016/j.envpol.2010.08.004

35. Hersikorn BD, Smits JEG. Compromised metamorphosis and thyroid hormone changes in wood frogs (*Lithobates sylvaticus*) raised on reclaimed wetlands on the Athabasca oil sands. *Environ Pollut Barking Essex 1987* (2011) **159**:596–601. doi:10.1016/j.envpol.2010.10.005

36. Bryer PJ, Elliott JN, Willingham EJ. The effects of coal tar based pavement sealer on amphibian development and metamorphosis. *Ecotoxicology* (2006) **15**:241–247. doi:10.1007/s10646-005-0055-z

37. Newman RA. Effects of changing density and food level on metamorphosis of a desert amphibian, *Scaphiopus couchii*. *Ecology* (1994) **75**:1085–1096. doi:10.2307/1939432

38. Blouin MS. Comparing bivariate reaction norms among species: time and size at metamorphosis in three species of Hyla (Anura: Hylidae). *Oecologia* (1992) **90**:288–293. doi:10.1007/BF00317188

39. Leips J, Travis J. Metamorphic responses to changing food levels in two species of Hylid frogs. *Ecology* (1994) **75**:1345–1356. doi:10.2307/1937459

40. Rudolf VHW, Rödel M-O. Phenotypic plasticity and optimal timing of metamorphosis under uncertain time constraints. *Evol Ecol* (2007) **21**:121–142. doi:10.1007/s10682-006-0017-9

41. Nicieza AG. Interacting effects of predation risk and food availability on larval anuran behaviour and development. *Oecologia* (2000) **123**:497–505. doi:10.1007/s004420000343

42. Denver RJ, Mirhadi N, Phillips M. Adaptive plasticity in amphibian metamorphosis: Response of *Scaphiopus hammondii* tadpoles to habitat Ddesiccation. *Ecology* (1998) **79**:1859–1872. doi:10.1890/0012-9658(1998)079[1859:APIAMR]2.0.CO;2

43. Eichler VB, Gray LS. The influence of environmental lighting on the growth and prometamorphic development of larval *Rana pipiens*. *Dev Growth Differ* (1976) **18**:177–182. doi:10.1111/j.1440-169X.1976.00177.x

44. Wright ML, Jorey T, Myers YM, Fieldstad ML, Paquette CM, Clark MB. Influence of photoperiod, day length, and feeding schedule on tadpole growth and development. *Dev Growth Differ* (1988) **30**: 315-323.

45. Edwards MLO, Pivorun EB. The effects of photoperiod and different dosages of melatonin on metamorphic rate and weight gain in *Xenopus laevis* tadpoles. *Gen Comp Endocrinol* (1991) **81**:28–38. doi:10.1016/0016-6480(91)90122-M

46. Crump ML. Effect of habitat drying on developmental time and size at metamorphosis in *Hyla pseudopuma*. *Copeia* (1989) **1989**:794. doi:10.2307/1445521

47. O'Regan SM, Palen WJ, Anderson SC. Climate warming mediates negative impacts of rapid pond drying for three amphibian species. *Ecology* (2014) **95**:845–855. doi:10.1890/13-0916.1

48. Hayes T, Chan R, Licht P. Interactions of temperature and steroids on larval growth, development, and metamorphosis in a toad (*Bufo boreas*). *J Exp Zool* (1993) **266**:206–215. doi:10.1002/jez.1402660306

49. Freitas MB, Brown CT, Karasov WH. Warmer temperature modifies effects of polybrominated diphenyl ethers on hormone profiles in leopard frog tadpoles (*Lithobates pipiens*). *Environ Toxicol Chem* (2017) **36**:120–127. doi:10.1002/etc.3506

50. Frieden E, Wahlborg A, Howard E. Temperature control of the response of tadpoles to triiodothyronine. *Nature* (1965) **205**:1173–1176. doi:10.1038/2051173a0

51. Fry AE. Effects of temperature on shortening of isolated *Rana pipiens* tadpole tail tips. *J Exp Zool* (1972) **180**:197–207. doi:10.1002/jez.1401800207

52. Grant KP, Licht LE. Effects of ultraviolet radiation on life-history stages of anurans from Ontario, Canada. *Can J Zool* (1995) **73**:2292–2301. doi:10.1139/z95-271

53. Pahkala M, Laurila A, Merilä J. Carry-over effects of ultraviolet-B radiation on larval fitness in *Rana temporaria*. *Proc R Soc B Biol Sci* (2001) **268**:1699–1706. doi:10.1098/rspb.2001.1725

54. Hornung MW, Degitz SJ, Korte LM, Olson JM, Kosian PA, Linnum AL, Tietge JE. Inhibition of Thyroid hormone release from cultured amphibian thyroid glands by methimazole, 6-propylthiouracil, and perchlorate. *Toxicol Sci* (2010) **118**:42–51. doi:10.1093/toxsci/kfq166

55. Tietge JE, Butterworth BC, Haselman JT, Holcombe GW, Hornung MW, Korte JJ, Kosian PA, Wolfe M, Degitz SJ. Early temporal effects of three thyroid hormone synthesis inhibitors in *Xenopus laevis*. *Aquat Toxicol* (2010) **98**:44–50. doi:10.1016/j.aquatox.2010.01.014

56. Yamauchi K, Prapunpoj P, Richardson SJ. Effect of diethylstilbestrol on thyroid hormone binding to amphibian transthyretins. *Gen Comp Endocrinol* (2000) **119**:329–339. doi:10.1006/gcen.2000.7528

57. Vandorpe G, Kühn ER. Estradiol-17β silastic implants in female *Rana ridibunda* depress thyroid hormone concentrations in plasma and the *in vitro* 5′-monodeiodination activity of kidney homogenates. *Gen Comp Endocrinol* (1989) **76**:341–345. doi:10.1016/0016-6480(89)90127-5

58. Wright ML, Proctor KL, Alves CD. Hormonal profiles correlated with season, cold, and starvation in *Rana catesbeiana* (bullfrog) tadpoles. *Comp Biochem Physiol C Pharmacol Toxicol Endocrinol* (1999) **124**:109–116. doi:10.1016/S0742-8413(99)00060-2

59. Boorse GC, Denver RJ. Endocrine mechanisms underlying plasticity in metamorphic timing in spadefoot toads. *Integr Comp Biol* (2003) **43**:646–657.

60. Gomez-Mestre I, Kulkarni S, Buchholz DR. Mechanisms and consequences of developmental acceleration in tadpoles responding to pond drying. *PLoS ONE* (2013) **8**: doi:10.1371/journal.pone.0084266

61. Freitas JS, Kupsco A, Diamante G, Felicio AA, Almeida EA, Schlenk D. Influence of temperature on the thyroidogenic effects of diuron and its metabolite 3,4-dca in tadpoles of the American bullfrog (*Lithobates catesbeianus*). *Environ Sci Technol* (2016) **50**:13095–13104. doi:10.1021/acs.est.6b04076
